# Supplementary material for: Global phenology maps reveal the drivers and effects of seasonal asynchrony
Source: Nature. 2025 Aug 27;645(8079):133–40. doi: 10.1038/s41586-025-09410-3 (PMC12408380; doi:10.1038/s41586-025-09410-3)
Supplement: Supplementary file 1 — Supplementary Tables 1–4 [file 41586_2025_9410_MOESM1_ESM.pdf]

nature portfolio

https://doi.org/10.1038/s41586-025-09410-3

Supplementary information

Global phenology maps reveal the drivers and effects of seasonal asynchrony

In the format provided by the authors and unedited

Supplementary Information: Tables: Global phenology maps reveal how seasonal asynchrony can shape evolution

Authors: Drew E. Terasaki Hart[1,2,3*], Thao-Nguyen Bui[1], Lauren Di Maggio[4], Ian J. Wang[1]

Affiliations:

[1]Department of Environmental Science, Policy, and Management, University of California, Berkeley CA, USA

[2]The Nature Conservancy, Arlington VA, USA

[3]CSIRO Environment, Brisbane, Queensland, Australia

[4]Department of Statistics, University of California, Berkeley CA, USA

*Corresponding author: Drew.TerasakiHart@csiro.au

Contents:

Tables S1 to S4

Supplementary Table 1: Synopsis of all datasets. This table summarizes all datasets used in this study, including a brief identifier (column 'Dataset'), a short explanation of its use ('Use'), a description of the dataset and how it was used ('Description'), and URLs or citations for the both the original data source and the source from which it was accessed for this work.

Dataset	Use	Description	Data source
MODIS surface reflectance (MCD43A4 v061)	land surface phenology (NIR_V)	Terra- and Aqua-derived, daily-resolution, 16-day temporal composite of nadir BRDF-adjusted surface reflectance at 500 m resolution; used to calculate NIR_V at ~5.5 km resolution and model long-term average LSP	https://lpdaac.usgs.gov/products/mcd43a4v061/, public GEE asset used from https://developers.google.com/earth-engine/datasets/catalog/MODIS_061_MCD43A4
OCO-2 sun-induced chlorophyll fluorescence (SIF)	land surface phenology (SIF)	semi-monthly SIF (weighted average of 757 and 771 nm wavelengths), interpolated across gaps between OCO-2 orbital swaths using an ANN, at ~5.5 km resolution; used to model long-term average LSP and assess NIR_V LSP results	data manually downloaded from https://daac.ornl.gov/VEGETATION/guides/Global_High_Res_SIF_OCO2.html
intertropical convergence zone (ITCZ) map	visualization of LSP mapping results	static map depicting of the mean position of the ITCZ in the summer, published as part of a review on the global monsoon system	digitized from Zhisheng, A. et al. Global Monsoon Dynamics and Climate Change. Annu Rev Earth Pl Sc 43, 1–49 (2015) (doi: 10.1146/annurev-earth-060313-054623) using WebPlotDigitizer version 4.6: https://automeris.io/WebPlotDigitizer
TROPOMI SIF	assessment of interpolated OCO-2 SIF dataset	ungridded SIF (at 740 nm wavelength) from TROPOMI sensor; used to assess the seasonality of the SIF values interpolated into OCO-2 orbital gaps across the pan-tropics	data manually downloaded from https://doi.org/10.22002/D1.1347
MODIS annual land cover (MCD12C1.061)	filtering of LSP datasets; phenological asynchrony driver	annual estimation of majority land cover type and sub-pixel proportions of land cover types, derived from Aqua and Terra data, classed according to the International Geosphere-Biosphere Programme's (IGBP) classification scheme ('Land Cover Type 1'), mapped at the ~5.5 km resolution of the Climate Modeling Grid (CMG); used to filter invalid land cover types out of LSP datasets and additionally filter agricultural land cover types out of LSP asynchrony datasets; also used to calculate entropy, in 100 km-radius circular neighborhoods, of vegetation structural types, for use in phenological asynchrony drivers analysis	https://lpdaac.usgs.gov/products/mcd12c1v061/, public GEE asset used from https://developers.google.com/earth-engine/datasets/catalog/MODIS_061_MCD12C1
PhenoCam normalized difference vegetation index (NDVI)	assessment of LSP map	time series of 'camera NDVI' (an NDVI metric calculated using a combination of near-synchronous RGB and infrared imagery captured by ground-based phenology cameras), calculated as 3-day summaries, for NDVI-eligible camera sites across the PhenoCam network that were available at the time of download (March 5, 2025) and that fell within valid land cover types	data accessed using the *phenocamapi* R package: https://github.com/PhenoCamNetwork/phenocamapi

FLUXNET gross primary productivity (GPP)	assessment of LSP map	time series of daily gross primary productivity (GPP) from all FLUXNET flux tower sites available at the time of download (October 11, 2021) and that fell within valid land cover types	data manually downloaded from https://fluxnet.org/data/
percent annual herbaceous cover	analysis of cheatgrass-related phenologies	raster at ~30 m resolution estimating percent annual herbaceous cover for the 2016-2018 time period across the sagebrush biome region of the North American Great Basin	data manually downloaded from https://doi.org/10.5066/P9VL3LD5
TerraClimate minimum temperature	phenological asynchrony driver	monthly minimum temperature, based on 64 years (01/01/1958 - 12/01/2021) of TerraClimate monthly means at ~4 km resolution; used to calculate spatial asynchrony of its seasonality	https://www.nature.com/articles/sdata2017191, public GEE asset used from https://developers.google.com/earth-engine/datasets/catalog/IDAHO_EPSCOR_TERRACLIMATE
TerraClimate maximum temperature	phenological asynchrony driver	monthly maximum temperature, based on 64 years (01/01/1958 - 12/01/2021) of TerraClimate monthly means at ~4 km resolution; used to calculate spatial asynchrony of its seasonality	https://www.nature.com/articles/sdata2017191, public GEE asset used from https://developers.google.com/earth-engine/datasets/catalog/IDAHO_EPSCOR_TERRACLIMATE
TerraClimate precipitation	phenological asynchrony driver	monthly precipitation, based on 64 years (01/01/1958 - 12/01/2021) of TerraClimate monthly means ~4 km resolution; used to calculate spatial asynchrony of its seasonality	https://www.nature.com/articles/sdata2017191, public GEE asset used from https://developers.google.com/earth-engine/datasets/catalog/IDAHO_EPSCOR_TERRACLIMATE
TerraClimate climate water deficit	phenological asynchrony driver	monthly climate water deficit, based on 64 years (01/01/1958 - 12/01/2021) of TerraClimate monthly means at 1/24°-resolution; used to calculate spatial asynchrony of its seasonality	https://www.nature.com/articles/sdata2017191, public GEE asset used from https://developers.google.com/earth-engine/datasets/catalog/IDAHO_EPSCOR_TERRACLIMATE
MODIS Aqua and Terra surface reflectance cloud bands	phenological asynchrony driver	10 years (01/01/2010 - 01/01/2020) of MODIS Aqua (MYD09GA v.006) and Terra (MOD09GA v.006) daily cloud band data; used to calculate daily average fractional cloud cover within ~5 km pixels	public GEE assets used from https://developers.google.com/earth-engine/datasets/catalog/MODIS_061_MYD09GA, https://developers.google.com/earth-engine/datasets/catalog/MODIS_061_MOD09GA
EarthEnv topographic complexity	phenological asynchrony driver	the Vector Ruggedness Metric, derived from GMTED2010 (Global Multi-resolution Terrain Elevation Data 2010) DEM (Digital Elevation Model) dataset, calculated as a median within ~100 km-resolution pixels; used to represent topographic complexity within a ~100 km neighborhood	data manually downloaded from http://www.earthenv.org/topography
Global Land Analysis & Discovery (GLAD) global land cover and land use 2019	phenological asynchrony driver	version 1 of a global, harmonized, map of land use and land cover in 2019, at ~30 m Landsat resolution; used to calculate the mean, within 100 km-radius circular neighborhoods, of the proportion of	https://glad.umd.edu/dataset/global-land-cover-land-use-v1; publicly accessible private GEE asset used from

		30 m pixels within a ~5.5 km analysis pixel that exhibits either human land use or a change in land use/cover most likely attributable to human activity	‘projects/glad/GLCmap2019’
MODIS monthly burned area (MCD64A1.v061)	phenological asynchrony driver	monthly burned area at ~500 m resolution, derived from Aqua and Terra 500 m surface reflectance and 1 km active fire observations; used to calculate the mean temporal frequency of burn events within 100 km-radius circular neighborhoods	https://lpdaac.usgs.gov/products/mcd64a1v061/, public GEE asset used from https://developers.google.com/earth-engine/datasets/catalog/MODIS_061_MCD64A1
WorldClim bioclimatic variables	isoclimatic phenology asynchrony analysis; allochrony by allopatry analyses	long-term averages, over the WorldClim version 2.1 1970-2000 time period, of 19 bioclimatic variables, mapped at 2.5 minutes resolution (~4.6 km); used to calculate pairwise climatic differences between sites	manually downloaded from https://www.worldclim.org/data/worldclim21.html
iNaturalist flowering observations	allochrony by allopatry analyses	histograms of flowering observation counts by week of year, and first ≤5000 records with individual flowering observation dates and locations, for all iNaturalist flowering plant taxa with at least 50 available native, non-captive, georeferenced, and research-grade flowering records	data accessed using the *pyinaturalist* python package: https://github.com/pyinat/pyinaturalist
Rhinella granulosa single nucleotide polymorphism (SNP) data	allochrony by allopatry analyses	location data and STRUCTURE-formatted allele frequency data for 7674 SNPs sequenced for 80 toads sampled from 51 sites	manually downloaded from the Dryad repository for Thomé et al. 2021: https://datadryad.org/stash/dataset/doi:10.5061/dryad.pc866t1p4
Xiphorhynchus fuscus cytochrome B sequence data	allochrony by allopatry analyses	location data and FASTA-formatted cytB sequence data for 34 samples from 22 sites	manually downloaded from the Zenodo repository for Quintero et al. 2014: http://zenodo.org/records/5012226, and from that study’s accessions on GenBank (accession records identified within the Zenodo repository)
Fedecafé Colombian coffee harvest season map	allochrony by allopatry analyses	static map depicting the delineation of the four official Colombian coffee harvest seasonalities, published by Federación Nacional de Cafeteros de Colombia	sampling points digitized from Ramírez Bacca, R. Mujeres en la caficultura tradicional colombiana, 1910-1970. Hist. Y Mem. 43–73 (2015) (doi:10.19053/20275137.3200) using WebPlotDigitizer version 4.6: https://automeris.io/WebPlotDigitizer

Supplementary Table 2: PhenoCam sites and LSP evaluation results. Camera site information and evaluation results for all PhenoCam NDVI datasets used for comparison with SIF and NIR_V LSP datasets. The vegetation type column ('Veg') lists all types that occur within all regions of interest (ROIs) masked within a camera's field of view and used to produce separate datasets. Type codes are: DB = deciduous broadleaf; DN = deciduous needleleaf; EB = evergreen broadleaf; EN = evergreen needleleaf; GR = grassland; MX = mixed vegetation (typically EN/DN, DB/EN, or DB/EB); SH = shrubs; TN = tundra; WL = wetland; NV = non-vegetated; UN,XX = unspecified. R^2 values indicate the percent variation in the phenocycle fitted to an LSP (NIR_V or SIF) dataset that is explained by the variation in a PhenoCam NDVI dataset's fitted annual cycle. Acknowledgments are provided as included in PhenoCam metadata.

PhenoCam camera site ID	Longitude	Latitude	Elevation	Veg	MAT (°C)	MAP (mm)	Start date	End date	R^2, NIR_V	R^2, SIF	Acknowledgments
ICOSFR-Fon1	2.7801	48.476336	115	DB	10.65	627	2022-01-01	2025-03-04	0.786	0.572	ICOS Research Infrastructure, CNRS, Université Paris-Saclay
NEON.D01.BART.DP1.00033	-71.287375	44.063869	285	DB; EN	5.93	1233	2016-12-13	2025-03-04	0.988	0.902	The NEON Data Usage and Citation Policy can be found at: http://data.neonscience.org/data-policy. NEON is a project sponsored by the National Science Foundation and operated under cooperative agreement by Battelle.
NEON.D01.BART.DP1.00042	-71.287375	44.063869	285	UN	5.93	1233	2016-12-13	2025-03-04	0.934	0.742	The NEON Data Usage and Citation Policy can be found at: http://data.neonscience.org/data-policy. NEON is a project sponsored by the National Science Foundation and operated under cooperative agreement by Battelle.
NEON.D01.HARV.DP1.00033	-72.17265	42.536911	359	DB; EN	7.12	1103	2016-12-13	2025-03-04	0.985	0.836	The NEON Data Usage and Citation Policy can be found at: http://data.neonscience.org/data-policy. NEON is a project sponsored by the National Science Foundation and operated under cooperative agreement by Battelle.
NEON.D01.HARV.DP1.00042	-72.17265	42.536911	359	UN	7.12	1103	2016-12-13	2025-03-04	0.958	0.786	The NEON Data Usage and Citation Policy can be found at: http://data.neonscience.org/data-policy. NEON is a

											project sponsored by the National Science Foundation and operated under cooperative agreement by Battelle.
NEON.D02.SCBI.DP1.00033	-78.139494	38.892925	364	DB	11.68	1027	2016-12-13	2025-03-04	0.986	0.901	The NEON Data Usage and Citation Policy can be found at: http://data.neonscience.org/data-policy. NEON is a project sponsored by the National Science Foundation and operated under cooperative agreement by Battelle.
NEON.D02.SERC.DP1.00033	-76.56001	38.89008	30	DB	13.17	1104	2016-01-29	2025-03-04	0.996	0.912	
NEON.D02.SERC.DP1.00042	-76.56001	38.89008	30	UN	13.17	1104	2016-02-23	2025-03-04	0.971	0.876	
NEON.D03.BARC.DP1.20002	-82.009089	29.676473	35	WL	20.44	1296	2017-10-30	2025-03-04	0.156	0.027	The NEON Data Usage and Citation Policy can be found at: http://data.neonscience.org/data-policy. NEON is a project sponsored by the National Science Foundation and operated under cooperative agreement by Battelle.
NEON.D03.DSNY.DP1.00033	-81.43619	28.12505	22	GR	22.38	1222	2016-12-14	2025-03-04	0.761	0.462	The NEON Data Usage and Citation Policy can be found at: http://data.neonscience.org/data-policy. NEON is a project sponsored by the National Science Foundation and operated under cooperative agreement by Battelle.
NEON.D03.DSNY.DP1.00042	-81.43619	28.12505	22	GR	22.38	1222	2016-12-14	2025-03-04	0.542	0.232	The NEON Data Usage and Citation Policy can be found at: http://data.neonscience.org/data-policy. NEON is a project sponsored by the National Science Foundation and operated under cooperative agreement by Battelle.
NEON.D03.JERC.DP1.00033	-84.468623	31.194839	58	DB; EN	19.15	1355	2016-12-14	2025-03-04	0.888	0.854	The NEON Data Usage and Citation Policy can be found at: http://data.neonscience.org/data-policy. NEON is a project sponsored by the National Science Foundation and operated

											under cooperative agreement by Battelle.
NEON.D03.JERC.DP1.00042	-84.468623	31.194839	58	UN	19.15	1355	2016-12-14	2025-03-04	0.937	0.917	The NEON Data Usage and Citation Policy can be found at: http://data.neonscience.org/data-policy. NEON is a project sponsored by the National Science Foundation and operated under cooperative agreement by Battelle.
NEON.D03.OSBS.DP1.00033	-81.993431	29.689282	56	EN	20.5	1290	2016-12-14	2025-03-04	0.414	0.207	The NEON Data Usage and Citation Policy can be found at: http://data.neonscience.org/data-policy. NEON is a project sponsored by the National Science Foundation and operated under cooperative agreement by Battelle.
NEON.D03.OSBS.DP1.00042	-81.993431	29.689282	56	GR	20.5	1290	2016-12-14	2025-03-04	0.801	0.463	The NEON Data Usage and Citation Policy can be found at: http://data.neonscience.org/data-policy. NEON is a project sponsored by the National Science Foundation and operated under cooperative agreement by Battelle.
NEON.D03.SUGG.DP1.20002	-82.016171	29.687054	28	WL	20.44	1296	2017-10-30	2025-03-04	0.527	0.485	The NEON Data Usage and Citation Policy can be found at: http://data.neonscience.org/data-policy. NEON is a project sponsored by the National Science Foundation and operated under cooperative agreement by Battelle.
NEON.D04.GUAN.DP1.00033	-66.8687	17.96955	136	EB	25.46	1027	2016-12-14	2025-03-04	0.003	0.118	The NEON Data Usage and Citation Policy can be found at: http://data.neonscience.org/data-policy. NEON is a project sponsored by the National Science Foundation and operated under cooperative agreement by Battelle.
NEON.D04.GUAN.DP1.00042	-66.8687	17.96955	136	UN	25.46	1027	2016-12-14	2025-03-04	0.045	0.123	The NEON Data Usage and Citation Policy can be found at: http://data.neonscience.org/data-policy. NEON is a project sponsored by the

											National Science Foundation and operated under cooperative agreement by Battelle.
NEON.D05.CRAM.DP1.20002	-89.47825	46.21111	518	WL	3.82	844	2017-10-30	2025-03-04	0.658	0.438	The NEON Data Usage and Citation Policy can be found at: http://data.neonscience.org/data-policy. NEON is a project sponsored by the National Science Foundation and operated under cooperative agreement by Battelle.
NEON.D05.STEI.DP1.00033	-89.58637	45.50894	476	DB; EN	4.69	803	2017-10-30	2025-03-04	0.934	0.983	The NEON Data Usage and Citation Policy can be found at: http://data.neonscience.org/data-policy. NEON is a project sponsored by the National Science Foundation and operated under cooperative agreement by Battelle.
NEON.D05.STEI.DP1.00042	-89.58637	45.50894	476	UN	4.69	803	2017-10-30	2025-03-04	0.998	0.873	The NEON Data Usage and Citation Policy can be found at: http://data.neonscience.org/data-policy. NEON is a project sponsored by the National Science Foundation and operated under cooperative agreement by Battelle.
NEON.D05.TREE.DP1.00033	-89.58572	45.49373	474	DB	4.76	803	2016-12-15	2025-03-04	0.988	0.941	The NEON Data Usage and Citation Policy can be found at: http://data.neonscience.org/data-policy. NEON is a project sponsored by the National Science Foundation and operated under cooperative agreement by Battelle.
NEON.D05.TREE.DP1.00042	-89.58572	45.49373	474	UN	4.76	803	2016-12-15	2025-03-04	0.993	0.859	The NEON Data Usage and Citation Policy can be found at: http://data.neonscience.org/data-policy. NEON is a project sponsored by the National Science Foundation and operated under cooperative agreement by Battelle.
NEON.D05.UNDE.DP1.00033	-89.537254	46.23391	529	DB	3.87	843	2016-12-14	2025-03-04	0.973	0.881	The NEON Data Usage and Citation Policy can be found at: http://data.neonscience.or

											g/data-policy. NEON is a project sponsored by the National Science Foundation and operated under cooperative agreement by Battelle.
NEON.D05.UNDE.DP1.00042	-89.537254	46.23391	529	UN	3.87	843	2016-12-14	2025-03-04	0.99	0.791	The NEON Data Usage and Citation Policy can be found at: http://data.neonscience.org/data-policy. NEON is a project sponsored by the National Science Foundation and operated under cooperative agreement by Battelle.
NEON.D06.KING.DP1.20002	-96.60336	39.10506	339	WL	12.46	888	2016-12-15	2025-03-04	0.991	0.934	The NEON Data Usage and Citation Policy can be found at: http://data.neonscience.org/data-policy. NEON is a project sponsored by the National Science Foundation and operated under cooperative agreement by Battelle.
NEON.D06.KONZ.DP1.00033	-96.56309	39.10077	415	GR	12.35	892	2017-02-24	2025-03-04	0.97	0.948	The NEON Data Usage and Citation Policy can be found at: http://data.neonscience.org/data-policy. NEON is a project sponsored by the National Science Foundation and operated under cooperative agreement by Battelle.
NEON.D06.KONZ.DP1.00042	-96.56309	39.10077	415	GR	12.35	892	2017-02-24	2025-03-04	0.966	0.93	The NEON Data Usage and Citation Policy can be found at: http://data.neonscience.org/data-policy. NEON is a project sponsored by the National Science Foundation and operated under cooperative agreement by Battelle.
NEON.D07.GRSM.DP1.00033	-83.50195	35.68896	589	DB	12.97	1367	2017-01-24	2025-03-04	0.98	0.889	The NEON Data Usage and Citation Policy can be found at: http://data.neonscience.org/data-policy. NEON is a project sponsored by the National Science Foundation and operated under cooperative agreement by Battelle.
NEON.D07.GRSM.DP1.00042	-83.50195	35.68896	589	UN	12.97	1367	2017-01-24	2025-03-04	0.989	0.911	The NEON Data Usage and Citation Policy can be

											found at: http://data.neonscience.org/data-policy. NEON is a project sponsored by the National Science Foundation and operated under cooperative agreement by Battelle.
NEON.D07.MLBS.DP1.00033	-80.524847	37.378314	1177	DB	9	1122	2016-12-15	2025-03-04	0.97	0.809	The NEON Data Usage and Citation Policy can be found at: http://data.neonscience.org/data-policy. NEON is a project sponsored by the National Science Foundation and operated under cooperative agreement by Battelle.
NEON.D07.MLBS.DP1.00042	-80.524847	37.378314	1177	UN	9	1122	2016-12-15	2025-03-04	0.996	0.893	The NEON Data Usage and Citation Policy can be found at: http://data.neonscience.org/data-policy. NEON is a project sponsored by the National Science Foundation and operated under cooperative agreement by Battelle.
NEON.D07.ORNL.DP1.00033	-84.282588	35.964128	351	DB	13.9	1220	2016-12-15	2025-03-04	0.99	0.91	The NEON Data Usage and Citation Policy can be found at: http://data.neonscience.org/data-policy. NEON is a project sponsored by the National Science Foundation and operated under cooperative agreement by Battelle.
NEON.D07.ORNL.DP1.00042	-84.282588	35.964128	351	UN	13.9	1220	2016-12-15	2025-03-04	0.952	0.807	The NEON Data Usage and Citation Policy can be found at: http://data.neonscience.org/data-policy. NEON is a project sponsored by the National Science Foundation and operated under cooperative agreement by Battelle.
NEON.D07.WALK.DP1.20002	-84.28041	35.95947	274	WL	13.9	1220	2016-06-29	2025-03-04	0.974	0.9	
NEON.D08.BLWA.DP1.20002	-87.798151	32.541529	23	DB	17.77	1400	2018-10-10	2025-03-04	0.902	0.722	The NEON Data Usage and Citation Policy can be found at: http://data.neonscience.org/data-policy. NEON is a project sponsored by the National Science Foundation and operated

											under cooperative agreement by Battelle.
NEON.D08.DELA.DP1.00033	-87.803877	32.541727	36	DB	17.72	1402	2016-12-16	2025-03-04	0.923	0.79	The NEON Data Usage and Citation Policy can be found at: http://data.neonscience.org/data-policy. NEON is a project sponsored by the National Science Foundation and operated under cooperative agreement by Battelle.
NEON.D08.DELA.DP1.00042	-87.803877	32.541727	36	UN	17.72	1402	2017-10-30	2025-03-04	0.919	0.889	The NEON Data Usage and Citation Policy can be found at: http://data.neonscience.org/data-policy. NEON is a project sponsored by the National Science Foundation and operated under cooperative agreement by Battelle.
NEON.D08.LENO.DP1.00033	-88.16122	31.85388	10	DB	18.12	1513	2016-05-06	2025-03-04	0.908	0.748	
NEON.D08.LENO.DP1.00042	-88.16122	31.85388	10	UN	18.12	1513	2017-10-30	2025-03-04	0.94	0.841	
NEON.D08.TALL.DP1.00033	-87.393259	32.95047	167	EN	16.88	1449	2016-12-16	2025-03-04	0.551	0.448	The NEON Data Usage and Citation Policy can be found at: http://data.neonscience.org/data-policy. NEON is a project sponsored by the National Science Foundation and operated under cooperative agreement by Battelle.
NEON.D08.TALL.DP1.00042	-87.393259	32.95047	167	UN	16.88	1449	2016-12-16	2025-03-04	0.974	0.836	The NEON Data Usage and Citation Policy can be found at: http://data.neonscience.org/data-policy. NEON is a project sponsored by the National Science Foundation and operated under cooperative agreement by Battelle.
NEON.D08.TOMB.DP1.20002	-88.158872	31.853431	10	DB; GR	18.12	1513	2018-07-24	2025-03-04	0.935	0.789	The NEON Data Usage and Citation Policy can be found at: http://data.neonscience.org/data-policy. NEON is a project sponsored by the National Science Foundation and operated under cooperative agreement by Battelle.

NEON.D09.NOGP.DP1.00033	-100.91535	46.76972	589	GR	5.72	428	2017-10-29	2025-03-04	0.971	0.933	The NEON Data Usage and Citation Policy can be found at: http://data.neonscience.org/data-policy. NEON is a project sponsored by the National Science Foundation and operated under cooperative agreement by Battelle.
NEON.D09.NOGP.DP1.00042	-100.91535	46.76972	589	GR	5.72	428	2017-10-31	2025-03-04	0.969	0.972	The NEON Data Usage and Citation Policy can be found at: http://data.neonscience.org/data-policy. NEON is a project sponsored by the National Science Foundation and operated under cooperative agreement by Battelle.
NEON.D10.ARIK.DP1.20002	-102.447103	39.758246	1188	GR	10.36	432	2016-12-19	2025-03-04	0.872	0.577	
NEON.D10.CPER.DP1.00033	-104.7456	40.81553	1600	GR	9.08	367	2016-06-29	2025-03-04	0.854	0.665	The NEON Data Usage and Citation Policy can be found at: http://data.neonscience.org/data-policy. NEON is a project sponsored by the National Science Foundation and operated under cooperative agreement by Battelle
NEON.D11.CLBJ.DP1.00033	-97.57	33.40123	279	DB	17.45	870	2017-02-13	2025-03-04	0.973	0.897	The NEON Data Usage and Citation Policy can be found at: http://data.neonscience.org/data-policy. NEON is a project sponsored by the National Science Foundation and operated under cooperative agreement by Battelle.
NEON.D11.OAES.DP1.00033	-99.05879	35.41059	520	GR	15.58	700	2017-02-27	2025-03-04	0.894	0.791	The NEON Data Usage and Citation Policy can be found at: http://data.neonscience.org/data-policy. NEON is a project sponsored by the National Science Foundation and operated under cooperative agreement by Battelle.
NEON.D11.OAES.DP1.00042	-99.05879	35.41059	520	GR	15.58	700	2017-02-27	2025-03-03	0.893	0.799	The NEON Data Usage and Citation Policy can be found at: http://data.neonscience.org/data-policy. NEON is a

											project sponsored by the National Science Foundation and operated under cooperative agreement by Battelle.
NEON.D12.BLDE.DP1.20002	-110.58715	44.95011	2035	GR	1.9	564	2018-09-25	2025-03-04	0.97	0.547	The NEON Data Usage and Citation Policy can be found at: http://data.neonscience.org/data-policy. NEON is a project sponsored by the National Science Foundation and operated under cooperative agreement by Battelle.
NEON.D12.YELL.DP1.00033	-110.53914	44.95348	2130	EN	1.5	590	2018-09-30	2025-03-04	0.745	0.925	The NEON Data Usage and Citation Policy can be found at: http://data.neonscience.org/data-policy. NEON is a project sponsored by the National Science Foundation and operated under cooperative agreement by Battelle.
NEON.D12.YELL.DP1.00042	-110.53914	44.95348	2130	UN	1.5	590	2018-10-10	2025-03-04	0.955	0.504	The NEON Data Usage and Citation Policy can be found at: http://data.neonscience.org/data-policy. NEON is a project sponsored by the National Science Foundation and operated under cooperative agreement by Battelle.
NEON.D13.MOAB.DP1.00033	-109.38827	38.24833	1803	GR	10.38	306	2017-02-24	2025-03-04	0.769	0.183	The NEON Data Usage and Citation Policy can be found at: http://data.neonscience.org/data-policy. NEON is a project sponsored by the National Science Foundation and operated under cooperative agreement by Battelle.
NEON.D13.MOAB.DP1.00042	-109.38827	38.24833	1803	GR	10.38	306	2017-02-24	2025-03-04	0.888	0.125	The NEON Data Usage and Citation Policy can be found at: http://data.neonscience.org/data-policy. NEON is a project sponsored by the National Science Foundation and operated under cooperative agreement by Battelle.
NEON.D13.NIWO.DP1.00033	-105.58237	40.05425	3493	TN	0.83	623	2017-04-25	2025-02-16	0.994	0.589	The NEON Data Usage and Citation Policy can be found at:

											http://data.neonscience.org/data-policy. NEON is a project sponsored by the National Science Foundation and operated under cooperative agreement by Battelle.
NEON.D13.NIWO.DP1.00042	-105.58237	40.05425	3493	TN	0.83	623	2017-03-09	2025-03-04	0.962	0.656	The NEON Data Usage and Citation Policy can be found at: http://data.neonscience.org/data-policy. NEON is a project sponsored by the National Science Foundation and operated under cooperative agreement by Battelle.
NEON.D14.JORN.DP1.00033	-106.84254	32.59068	1321	GR	15.18	273	2017-02-24	2025-03-04	0.289	0.219	The NEON Data Usage and Citation Policy can be found at: http://data.neonscience.org/data-policy. NEON is a project sponsored by the National Science Foundation and operated under cooperative agreement by Battelle. This research was a contribution from the Long-Term Agroecosystem Research (LTAR) network. LTAR is supported by the United States Department of Agriculture.
NEON.D14.JORN.DP1.00042	-106.84254	32.59068	1321	GR	15.18	273	2017-02-24	2025-03-04	0.827	0.203	The NEON Data Usage and Citation Policy can be found at: http://data.neonscience.org/data-policy. NEON is a project sponsored by the National Science Foundation and operated under cooperative agreement by Battelle. This research was a contribution from the Long-Term Agroecosystem Research (LTAR) network. LTAR is supported by the United States Department of Agriculture.
NEON.D14.SRER.DP1.00033	-110.83549	31.91068	999	SH	19.16	395	2017-02-24	2025-03-04	0.696	0.789	The NEON Data Usage and Citation Policy can be found at: http://data.neonscience.org/data-policy. NEON is a project sponsored by the National Science Foundation and operated under cooperative agreement by Battelle.

NEON.D14.SRER.DP1.00042	-110.83549	31.91068	999	SH	19.16	395	2017-02-24	2025-03-04	0.851	0.917	The NEON Data Usage and Citation Policy can be found at: http://data.neonscience.org/data-policy. NEON is a project sponsored by the National Science Foundation and operated under cooperative agreement by Battelle.
NEON.D15.ONAQ.DP1.00033	-112.452452	40.177599	1655	GR; SH	9.41	297	2016-12-19	2025-03-04	0.953	0.541	The NEON Data Usage and Citation Policy can be found at: http://data.neonscience.org/data-policy. NEON is a project sponsored by the National Science Foundation and operated under cooperative agreement by Battelle.
NEON.D15.ONAQ.DP1.00042	-112.452452	40.177599	1655	GR; SH	9.41	297	2016-12-19	2025-03-04	0.843	0.4	The NEON Data Usage and Citation Policy can be found at: http://data.neonscience.org/data-policy. NEON is a project sponsored by the National Science Foundation and operated under cooperative agreement by Battelle.
NEON.D16.ABBY.DP1.00033	-122.33033	45.76243	390	EN	9.13	2372	2017-05-30	2025-03-04	0.633	0.26	The NEON Data Usage and Citation Policy can be found at: http://data.neonscience.org/data-policy. NEON is a project sponsored by the National Science Foundation and operated under cooperative agreement by Battelle.
NEON.D16.ABBY.DP1.00042	-122.33033	45.76243	390	UN	9.13	2372	2017-10-31	2025-03-04	0.852	0.506	The NEON Data Usage and Citation Policy can be found at: http://data.neonscience.org/data-policy. NEON is a project sponsored by the National Science Foundation and operated under cooperative agreement by Battelle
NEON.D16.WREF.DP1.00033	-121.95191	45.82049	368	EN	9.05	1966	2018-02-01	2025-03-04	0.978	0.75	The NEON Data Usage and Citation Policy can be found at: http://data.neonscience.org/data-policy. NEON is a project sponsored by the National Science Foundation and operated under cooperative

											agreement by Battelle.
NEON.D16.WREF.DP1.00042	-121.95191	45.82049	368	UN	9.05	1966	2018-02-01	2025-02-23	0.838	0.628	The NEON Data Usage and Citation Policy can be found at: http://data.neonscience.org/data-policy. NEON is a project sponsored by the National Science Foundation and operated under cooperative agreement by Battelle.
NEON.D17.SJER.DP1.00042	-119.73228	37.10878	398	GR	16.67	542	2018-07-12	2025-03-04	0.952	0.99	The NEON Data Usage and Citation Policy can be found at: http://data.neonscience.org/data-policy. NEON is a project sponsored by the National Science Foundation and operated under cooperative agreement by Battelle.
NEON.D17.SOAP.DP1.00042	-119.26219	37.03337	1210	UN	13.3	842	2017-11-02	2025-03-04	0.849	0.481	The NEON Data Usage and Citation Policy can be found at: http://data.neonscience.org/data-policy. NEON is a project sponsored by the National Science Foundation and operated under cooperative agreement by Battelle.
NEON.D17.TEAK.DP1.00033	-119.00602	37.00583	2149	EN	6.17	831	2018-07-24	2025-03-04	0.723	0.737	The NEON Data Usage and Citation Policy can be found at: http://data.neonscience.org/data-policy. NEON is a project sponsored by the National Science Foundation and operated under cooperative agreement by Battelle.
NEON.D17.TEAK.DP1.00042	-119.00602	37.00583	2149	UN	6.17	831	2018-07-24	2025-03-03	0.95	0.531	The NEON Data Usage and Citation Policy can be found at: http://data.neonscience.org/data-policy. NEON is a project sponsored by the National Science Foundation and operated under cooperative agreement by Battelle.
NEON.D20.PUUM.DP1.00033	-155.31731	19.55309	1683	EB	13.24	2155	2019-04-25	2025-01-25	0.045	0.178	The NEON Data Usage and Citation Policy can be found at: http://data.neonscience.org/data-policy. NEON is a project sponsored by the National Science

											Foundation and operated under cooperative agreement by Battelle.
NEON.D20.PUUM.DP1.00042	-155.31731	19.55309	1683	UN	13.24	2155	2019-04-25	2025-01-25	0.334	0.282	The NEON Data Usage and Citation Policy can be found at: http://data.neonscience.org/data-policy. NEON is a project sponsored by the National Science Foundation and operated under cooperative agreement by Battelle.
alercecosteroforest	-73.4439	-40.1726	840	EN	7.39	1914	2023-01-24	2025-03-04	0.184	0.045	National Agency of Research and Development (ANID, Chile), grants FONDECYT 1211652 and FONDAP 15110009
alligatorriver	-75.9038	35.7879	1	DB	16.13	1327	2012-05-03	2021-12-21	0.971	0.933	Research at the Alligator River flux site is supported by DOE NICCR (award 08-SC-NICCR-1072), DOE-TES (awards 11-DE-SC-0006700 and 7090112), USDA Forest Service (award 13-JV-11330110-081) and USDA-NIFA (award 2014-67003-22068).
anisclo	0.050894316	42.55940876	952	EB	8.62	961	2023-04-18	2024-11-25	0.964	0.811	European Commission – NextGenerationEU (Regulation EU 2020/2094), through CSICs Global Health Platform (PTI Salud Global)
arbutuslakeinlet	-74.24527	43.99336	527	DB; EN	4.71	1074	2015-08-19	2025-03-02	0.957	0.908	Research at this site is supported by the New York State Energy Research and Development Authority (NYSERDA).
arsgreatbasinltar098	-116.7132	43.1675	1426	SH	8.46	309	2017-05-18	2025-03-04	0.918	0.927	This research at the Reynolds Creek Experimental Watershed and the Great Basin Long-Term Agroecosystem Research (LTAR) site is funded by the USDA Agricultural Research Service (ARS) through ARS Project Numbers 2052-13610-011-00-D and 2052-13610-012-00-D and is a collaborative contribution to the LTAR Network. This camera

											location occurs on rangelands managed by the USDI Bureau of Land Management and is also co-located on a site within the Reynolds Creek Critical Zone Observatory which is funded by the National Science Foundation under Grant Number EAR-1331872. This research was a contribution from the Long-Term Agroecosystem Research (LTAR) network. LTAR is supported by the United States Department of Agriculture.
arsgreatbasinltar117	-116.7357	43.1432	1625	SH	7.15	375	2021-03-10	2025-03-04	0.725	0.816	This research at the Reynolds Creek Experimental Watershed and the Great Basin Long-Term Agroecosystem Research (LTAR) site is funded by the USDA Agricultural Research Service (ARS) through ARS Project Numbers 2052-13610-011-00-D and 2052-13610-012-00-D and is a collaborative contribution to the LTAR Network. This camera location occurs on rangelands managed by the USDI Bureau of Land Management and is also co-located on a site within the Reynolds Creek Critical Zone Observatory which is funded by the National Science Foundation under Grant Number EAR-1331872. This research was a contribution from the Long-Term Agroecosystem Research (LTAR) network. LTAR is supported by the United States Department of Agriculture.
arsgreatbasinltar177	-116.7484	43.0645	2114	SH	5.2	448	2018-10-17	2025-03-04	0.637	0.138	This research at the Reynolds Creek Experimental Watershed and the Great Basin Long-Term Agroecosystem Research (LTAR) site is funded by the USDA Agricultural Research Service (ARS) through ARS Project Numbers 2052-13610-011-00-D and 2052-13610-012-00-D and

											is a collaborative contribution to the LTAR Network. This camera location occurs on rangelands managed by the USDI Bureau of Land Management and is also co-located on a site within the Reynolds Creek Critical Zone Observatory which is funded by the National Science Foundation under Grant Number EAR-1331872. This research was a contribution from the Long-Term Agroecosystem Research (LTAR) network. LTAR is supported by the United States Department of Agriculture.
austincary	-82.2188	29.738	44	EN	20.28	1307	2016-01-11	2021-06-28	0.288	0.371	
barrocolorado	-79.8475	9.1582	150	MX	26.42	2394	2013-03-21	2025-03-04	0.016	0.06	
barrocolorado4	-79.8482	9.1565	164	EB	26.42	2394	2019-01-29	2025-02-14	0.264	0.113	
bartlettir	-71.2881	44.0646	268	DB; EN	5.93	1233	2008-04-18	2025-03-04	0.997	0.922	Research at the Bartlett Experimental Forest tower is supported by the National Science Foundation (grant DEB-1114804) and the USDA Forest Service's Northern Research Station.
bbc1	-72.174359	42.53508	362	DB	7.12	1103	2015-05-04	2025-03-04	0.966	0.88	
bezamahafaly	44.6289	-23.6558	165	DB; EB	24.52	651	2023-07-02	2025-03-04	0.347	0.136	Ankoatsifaka Initiative for Dry Forests, 501c3
bitterbrush001	-119.9456	48.154	667	GR	7.44	359	2020-09-20	2024-10-12	0.904	0.795	Research at this site is supported by GSI Environmental Inc. (http://www.gsi-net.com), Olympia, WA.
blackrockforest	-74.0239	41.3974	427	DB	9.16	1309	2020-05-22	2024-10-11	0.99	0.875	CUNY Advanced Science Research Center; Black Rock Forest Consortium
blueoakheadquarters	-121.7393	37.3827	572	DB; GR	14.29	826	2022-01-24	2025-03-04	0.728	0.55	Blue Oak Ranch Reserve is part of the University of California Natural Reserve System. Blue Oak Ranch is administered bu the University of California,

											Berkeley
boglakepeatland	-93.4893	47.5051	416	WL	3.59	695	2021-03-18	2025-03-04	0.931	0.673	USDA-USFS
bozeman	-110.777778	45.783056	2332	EN; GR	2.97	560	2015-08-16	2019-09-13	0.994	0.851	Research at the Bozeman site is supported by Colorado State University and the AmericaView program (grants G13AC00393, G11AC20461, G15AC00056) with phenocam equipment and deployment sponsored by the Department of Interior North Central Climate Science Center.
brackishimpoundment	-79.2973	33.2015	1	WL	18.13	1350	2020-03-20	2025-03-04	0.446	0.723	Clemson University College of Agriculture, Forestry and Life Sciences
bullshoals	-93.06663	36.562833	260	DB; GR	14.21	1084	2013-11-20	2025-03-04	0.998	0.927	
burnssagebrush	-119.6909	43.4712	1398	SH	7.39	269	2012-10-12	2022-09-09	0.849	0.305	This site was supported by the NOAA Earth System Science Program (Grant number NOAA-OAR-CPO-2012-2003041).
canadaOBS	-105.11779	53.98717	628	DN; EN	0.03	475	2011-07-05	2025-03-04	0.975	0.848	BERMS sites are funded through the Global Institute for Water Security (GIWS) at the University of Saskatchewan
canadaoa2	-106.19779	53.62889	601	DB	0.65	465	2016-06-16	2018-07-21	0.976	0.922	
cedarmesa	-109.7471	37.5241	1860	EN; GR; SH; XX	10.79	297	2023-05-23	2025-02-25	0.851	0.023	Colorado River Authority of Utah; University of Utah; Utah Geological Survey; Utah School and Institutional Trust Lands Administration
centralredwoods1	-123.301511	38.632272	200	EN	12.57	1080	2020-06-28	2025-03-04	0.861	0.78	
centralredwoods2	-123.301511	38.632272	200	EN	12.57	1080	2021-10-09	2025-03-04	0.917	0.924	
coaloilpoint1	-119.880226	34.413695	6	GR	15.84	423	2022-10-18	2025-03-04	0.936	0.954	
congoflux	24.5024	0.8144	357	EB	24.28	1774	2022-	2025-	0.491	0.072	

							05-10	03-04			
coweeta	-83.4275	35.0592	680	DB	12.07	1808	2011-04-08	2025-03-04	0.984	0.848	Research at the Coweeta flux tower is funded through the USDA Forest Service, Southern Research Station; USDA Agriculture and Food Research Initiative Foundational Program, award number 2012-67019-19484; EPA agreement number 13-IA-11330140-044; and the National Science Foundation, Long-Term Ecological Research (LTER) program, award #DEB-0823293.
cperheavy	-104.7386	40.8153	1652	GR	9.08	367	2018-05-24	2022-03-15	0.839	0.636	This research was a contribution from the Long-Term Agroecosystem Research (LTAR) network. LTAR is supported by the United States Department of Agriculture.
cperuvb	-104.7558542	40.8055764	1641	GR	9.03	369	2015-07-15	2025-03-04	0.902	0.629	Research at the cperuvb site is supported by Colorado State University and the AmericaView program (grants G13AC00393, G11AC20461, G15AC00056) with phenocam equipment and deployment sponsored by the Department of Interior North Central Climate Science Center.<br>This research was a contribution from the Long-Term Agroecosystem Research (LTAR) network. LTAR is supported by the United States Department of Agriculture.
czdryredlake	-106.8303	32.7122	1311	NV	15	271	2022-08-04	2025-03-04	0.473	0.07	The project is part of the Critical Zone Collaborative Network funded by NSF (Award Number 2012475). The Dryland CZ project includes collaborators at Boise State University, New Mexico State University, the University of Wyoming, and Insights El Paso. The project began on September 1, 2020.Red Lake Playa is located on the Jornada Experimental Range funded by NSF LTER and

											USDA-ARS.
dangermondbunkerhill	-120.4635	34.4909	5	GR	14.72	451	2022-02-03	2025-03-04	0.824	0.871	The Nature Conservancy
dangermondjalama	-120.458	34.5163	142	GR	14.56	489	2022-02-02	2025-03-04	0.818	0.871	The Nature Conservancy
delnortecounty1	-124.1	41.6	28	EN	11.49	2016	2020-03-12	2025-03-04	0.852	0.763	
delnortecounty2	-124.1	41.6	28	EN	11.49	2016	2020-03-12	2025-02-21	0.909	0.707	
donanafuenteduque	-6.434595	36.998553	1	WL	17.96	511	2017-11-11	2025-03-04	0.971	0.723	This site is maintained by the ICTS Doñana Scientific Reserve (ICTS-2009-39) funded by Spanish Ministry of Science, Innovation and Universities. Phenocam camera was acquired by funding from the Structural Project "Adaptation and improvement of the ICTS e-Infrastructure ICTS-RBD for LifeWatch (FEDER FICTS-2014/01/AIC-A-2011-0706)
donanapajarera	-6.4432	36.9962	2	DB	17.96	511	2017-11-11	2025-03-04	0.451	0.021	This site is maintained by the ICTS Doñana Scientific Reserve (ICTS-2009-39) funded by Spanish Ministry of Science, Innovation and Universities. Phenocam camera was acquired by funding from the Structural Project "Adaptation and improvement of the ICTS e-Infrastructure ICTS-RBD for LifeWatch (FEDER FICTS-2014/01/AIC-A-2011-0706)
dukehw	-79.100371	35.973583	400	DB	14.78	1165	2013-05-31	2025-03-04	0.987	0.9	
elverde	-65.8199	18.320654	312	EB	21.87	2048	2014-11-12	2023-12-11	0.315	0.469	Luquillo Long-Term Ecological Research is supported by the University of Puerto Rico, Natural Science Faculty, Department of Environmental Science and the National Science Foundation under Grants DEB-9705814 , DEB-0080538 , DEB-0218039 , BSR-8811902 , DEB-9411973 , DEB-0620910 , DEB-1239764, DEB-

											1546686.
eslm1	-5.7786833	39.9426889	265	EB; GR	15.86	465	2014-06-21	2025-02-25	0.918	0.82	We acknowledge the Alexander von Humboldt Foundation for supporting this research with the Max-Planck Prize to Markus Reichstein.
eslm2	-5.775881	39.934592	269	EB; GR	15.86	465	2014-06-23	2025-02-24	0.652	0.487	
eslma	-5.7746	39.9403	265	EB; GR	15.86	465	2015-01-31	2025-02-25	0.333	0.311	We acknowledge the GEISpain (CGL2014-52838-C2-2-R; MINECO) and ELEMENTAL (CGL2017-83538-C3-3-R, MINECO) projects, and the Alexander von Humboldt Foundation for supporting this research with the Max-Planck Prize to Markus Reichstein.
eucflux	-48.728009	-22.967875	750	EB	19.6	1272	2017-11-26	2025-03-04	0.101	0	CIRAD (Centre de Coopération Internationale en Recherche Agronomique pour le Développement); the EUCFLUX project (funded by Cenibra, Duratex, Eldorado, International Paper, Klabin, Suzano, Fibria through IPEF http://www.ipef.br/eucflux/en/); SOERE F-ORE-T which is supported annually by Ecofor, Allenvi and the French national research infrastructure ANAEE-F (http://www.anaee-france.fr/fr/); we acknowledge support from the IN-SYLVA French network.
fairmiemingaut2	10.9703	47.3166	960	SH	7.37	1150	2023-05-22	2025-03-04	0.632	0.638	Infrastructure grants by the University of Innsbruck via the research area Mountain Regions
flagstaffchimneysprings	-111.678542	35.257296	2250	EN; GR	6.52	550	2020-09-02	2025-03-04	0.929	0.667	Temuulen Sankey. 2020. Post-restoration ponderosa pine forest structure. Funded by Salt River Project and the Nature Conservancy. Associated Publication to cite: Belmonte, A., T. Sankey, J. Biedermann, J. Bradford, S. Goetz, T. Kolb, and T. Woolley. UAV-derived estimates of forest

											structure to inform ponderosa pine forest restoration. 2019. Remote Sensing in Ecology and Conservation 6: 181-197
forbes	-121.2972	39.232	360	GR	16.61	855	2017-01-25	2023-06-30	0.616	0.675	Project: A data-driven decision support system for monitoring and predicting forage production in California's rangeland sponsored by RUSSELL L. RUSTICI RANGELAND AND CATTLE RESEARCH ENDOWMENT
gcejuncus	-81.275	31.3868	4	WL	19.57	1313	2022-02-01	2025-03-04	0.117	0.322	Georgia Coastal Ecosystems LTER program, supported by NSF grant OCE-1832178
glees	-106.2394	41.3644	3150	EN	0.49	510	2015-11-11	2025-03-04	0.304	0.034	
goriz	0.015	42.6634	2200	GR	3.2	1342	2022-11-01	2024-02-18	0.934	0.847	European Commission – NextGenerationEU (Regulation EU 2020/2094), through CSICs Global Health Platform (PTI Salud Global)
grandteton	-110.578036	43.915017	2054	SH	1.87	645	2015-07-29	2021-01-29	0.441	0.803	This site has been supported by AmericaView, the USGS, Iowa State University, Montana State University, and the DOI North Central Climate Science Center.
grca1pj	-112.2211	36.0241	1978	EN	8.49	387	2017-08-20	2025-03-01	0.885	0.66	United States Geological Survey: Western Geographic Science Center; National Park Service: Inventory and Monitoring Program, Southern Colorado Plateau Network
greenridge1	-78.4067	39.6905	285	DB	10.67	952	2016-06-17	2023-12-06	0.914	0.764	The Green Ridge phenocam on the abandoned Town Hill firetower is logistically supported by the Maryland Department of Natural Resources, Green Ridge State Forest, and financially supported by West Virginia University internal grants to Brenden McNeil in the Department of Geology and Geography.

haha	-158.17	21.37	865	UN			2017-10-21	2025-03-04	0.004	0.142	Grant funding: Center for Conservation Research and Training (CCRT) Pacific Island Climate Change Cooperative (PICCC) and USGS Pacific Island Ecosystems Research Center (PIERC). Acknowledgements: Susan Ching and James Harmon (Department of Land and Natural Resources Division of Forestry and Wildlife (DLNR/DOFAW), Lauren Weisenberger (US Fish and Wildlife), Department of Geography (University of Hawaii at Manoa), Andrew Richardson (Northern Arizona University).
hanfordhn1	-119.275	46.4089	119	GR; SH	12.34	192	2023-11-09	2025-03-04	0.584	0.739	This research was supported by the U.S. Department of Energy (DOE), Office of Biological and Environmental Research (BER), as part of BERs Subsurface Biogeochemistry Research Program (SBR).
hartheim1	7.5981	47.9338	201	EN	10.57	797	2018-11-05	2025-03-04	0.978	0.86	Albert-Ludwigs-Universität Freiburg
hartheim2	7.5981	47.9338	201	UN	10.57	797	2018-11-05	2025-03-04	0.947	0.901	Albert-Ludwigs-Universität Freiburg
hartheim3	7.6006	47.9367	201	DB; GR	10.57	797	2020-09-10	2025-03-04	0.826	0.993	University of Freiburg, Germany
hartprairie	-111.7324	35.3536	2585	GR	4.63	614	2015-07-07	2017-03-30	0.353	0.729	Research at Hart Prairie is supported by the U.S. Geological Survey
harvardbarn	-72.1899	42.53532	350	DB; EN	7.12	1103	2011-07-27	2025-03-04	0.973	0.809	Research at Harvard Forest is partially supported through the National Science Foundation’s LTER program (DEB-1237491).
harvardems2	-72.1715	42.5378	340	DB	7.12	1103	2021-04-12	2025-03-04	0.988	0.846	The Harvard EMS site is supported is an AmeriFlux core site supported by the AmeriFlux Management Project with funding by the U.S. Department of Energy’s Office of Science under Contract No. DE-AC02-05CH11231, and a part of the Harvard Forest LTER site supported by

											the National Science Foundation (DEB-1237491).
harvardhemlock2	-72.177976	42.539407	355	EN	7.12	1103	2015-09-03	2025-03-04	0.246	0.1	Research at Harvard Forest is partially supported through the National Science Foundation’s LTER program (DEB-1237491).
harvardlph	-72.185	42.542	380	DB	7.09	1104	2010-06-15	2019-09-13	0.97	0.808	Research at Harvard Forest is partially supported through the National Science Foundation’s LTER program (DEB-1237491).
hobcawclearcutlongleaf	-79.232	33.3486	4	EN	18.01	1370	2018-12-18	2025-03-04	0.697	0.339	
hobcawlongleafunderstory	-79.244	33.3242	5	UN	17.98	1370	2024-01-27	2025-03-04	0.627	0.801	DOE: Improving models of stand and watershed carbon and water fluxes with more accurate representations of soil-plant-water dynamics in southern pine ecosystems
hobcawmaturelongleaf	-79.2439	33.3242	8	EN	17.98	1370	2018-12-17	2025-03-04	0.115	0.347	
honouliuli	-158.15	21.37	870	EB	23.71	805	2017-09-27	2025-03-04	0.638	0.166	Grant funding: Center for Conservation Research and Training (CCRT) Pacific Island Climate Change Cooperative (PICCC) and USGS Pacific Island Ecosystems Research Center (PIERC). Acknowledgements: Susan Ching and James Harmon (Department of Land and Natural Resources Division of Forestry and Wildlife (DLNR/DOFAW), Lauren Weisenberger (US Fish and Wildlife), Department of Geography (University of Hawaii at Manoa), Andrew Richardson (Northern Arizona University).
howland2	-68.7418	45.2128	79	DB	5.78	1062	2008-03-30	2022-01-08	0.917	0.757	Research at Howland Forest is supported by the Office of Science (BER), US Department of Energy, and the USDA Forest Service's Northern Research Station.
hubbardbrook	-71.701	43.9438	253	DB	6.24	1047	2008-	2025-	0.997	0.898	Research at the Hubbard

							04-16	03-04			Brook Experimental Forest is partially supported by the National Science Foundation's LTER program (DEB-1114804, DEB-1637685) and the USDA Forest Service Northern Research Station.
huyckpreserveny	-74.1587	42.5266	478	DB; EN	6.65	1035	2015-04-06	2025-03-04	0.977	0.945	Research at the preserve is supported by the NSF Award #145544: Collaborative Research: IDBR: TYPE A. The NANAPHID: A novel aphid-like nanosensor network for real-time measurements of carbohydrates in live plant tissue, and the NSF MRI Award #72205: Acquisition of a Small Unmanned Aircraft System of Natural and Urban Ecosystem Studies and Risk Disaster Management
ibp	-106.847	32.589	1325	GR; SH; XX	15.18	273	2013-05-10	2025-03-04	0.89	0.025	This research at the Jornada Experimental Range is funded by the USDA-Agriculture Research Service (ARS) via ARS Project Number 3050-11210-007-00D. Select camera locations are co-located with sites funded by National Science Foundation under Grant number DEB 1235828 as part of the Jornada Basin LTER program to New Mexico State University. This research was a contribution from the Long-Term Agroecosystem Research (LTAR) network. LTAR is supported by the United States Department of Agriculture.
it25matschb3	10.5919	46.6917	1930	EN; GR	4.64	548	2021-08-05	2025-03-04	0.863	0.454	Eurac Research: project open air laboratory LTSER Matsch/Mazia; Funding: Autonomous Province of Bolzano
it25matschf6	10.648	46.6955	2360	DN; EN; GR	0.9	634	2018-10-11	2025-03-04	0.918	0.744	Eurac Research: project open air laboratory LTSER Matsch/Mazia; Funding: Autonomous Province of Bolzano
it25matschs3	10.7108	46.7667	2680	TN	-4.76	1125	2016-	2025-	0.9	0.815	Eurac Research: project

							11-01	03-04			open air laboratory LTSER Matsch/Mazia; Funding: Autonomous Province of Bolzano
it25matschs4	10.6023	46.707	2400	TN	4.64	548	2016-10-27	2025-03-04	0.9	0.898	Eurac Research: project open air laboratory LTSER Matsch/Mazia; Funding: Autonomous Province of Bolzano
jasperridge	-122.22104	37.402028	197	GR	13.9	603	1999-12-31	2025-03-04	0.527	0.545	Supported by Jasper Ridge Biological Preserve of Stanford University and the Carnegie Institution Department of Global Ecology
jerbajada	-106.633793	32.57992	1402	SH	15.01	283	2014-04-21	2025-03-04	0.888	0.294	This research at the Jornada Experimental Range is funded by the USDA-Agriculture Research Service (ARS) via ARS Project Number 3050-11210-007-00D. Select camera locations are co-located with sites funded by National Science Foundation under Grant number DEB 1235828 as part of the Jornada Basin LTER program to New Mexico State University.<br>This research was a contribution from the Long-Term Agroecosystem Research (LTAR) network. LTAR is supported by the United States Department of Agriculture.
jergrassland	-106.8333	32.5833	1331	GR; XX	15.28	273	2019-02-18	2025-03-04	0.772	0.111	This research was a contribution from the Long-Term Agroecosystem Research (LTAR) network. LTAR is supported by the United States Department of Agriculture.
jergrassland2	-106.8261	32.5849	1328	GR; SH; XX	15.06	276	2022-01-22	2025-03-04	0.704	0.041	This research was a contribution from the Long-Term Agroecosystem Research (LTAR) network. LTAR is supported by the United States Department of Agriculture.
jernort	-106.788	32.619	1328	NV; SH; XX	15.03	276	2014-03-04	2025-03-04	0.968	0.17	This research at the Jornada Experimental Range is funded by the USDA-Agriculture Research Service (ARS) via ARS Project Number

											3050-11210-007-00D. Select camera locations are co-located with sites funded by National Science Foundation under Grant number DEB 1235828 as part of the Jornada Basin LTER program to New Mexico State University.
jernovel	-106.8334	32.6335	1324	GR; SH; XX	15.07	274	2019-02-13	2025-03-04	0.977	0.686	This research was a contribution from the Long-Term Agroecosystem Research (LTAR) network. LTAR is supported by the United States Department of Agriculture.
jernovel2	-106.8333	32.6465	1330	SH; XX	14.97	276	2022-01-22	2025-03-04	0.851	0.422	This research was a contribution from the Long-Term Agroecosystem Research (LTAR) network. LTAR is supported by the United States Department of Agriculture.
jernwern	-106.7387	32.6271	1325	NV; SH; XX	14.95	276	2017-11-06	2025-03-04	0.943	0.333	This research was a contribution from the Long-Term Agroecosystem Research (LTAR) network. LTAR is supported by the United States Department of Agriculture.
jersand	-106.798	32.515	1363	SH	15.34	285	2014-02-28	2025-03-04	0.96	0.438	This research at the Jornada Experimental Range is funded by the USDA-Agriculture Research Service (ARS) via ARS Project Number 3050-11210-007-00D. Select camera locations are co-located with sites funded by National Science Foundation under Grant number DEB 1235828 as part of the Jornada Basin LTER program to New Mexico State University.
jershrubland	-106.8334	32.6502	1328	SH; XX	15.07	274	2019-02-13	2025-03-04	0.979	0.302	USDA Agricultural Research Service LTAR network
jershrubland2	-106.831	32.6638	1336	SH; XX	14.97	276	2022-01-22	2025-03-04	0.748	0.08	This research was a contribution from the Long-Term Agroecosystem Research (LTAR) network. LTAR is supported by the United States Department of Agriculture.

junipersavannah	-105.8615	34.4254	1931	GR; SH	11.37	344	2020-10-27	2025-03-04	0.854	0.538	AmeriFlux
kamuela2	-155.661139	20.014959	830	GR	18.58	1624	2018-12-15	2025-03-04	0.894	0.012	The Kamuela PhenoCam is made possible by logistical support and internet access provided by Kiki Brown, with additional thanks to the Board of Directors at Holo Holo Ku.
kempnrs	-89.67755	45.83992	539	DB	4.32	804	2021-05-18	2025-03-04	0.972	0.919	University of Wisconsin - Madison
kendall	-109.94185	31.73652	1529	GR; SH	16.51	410	2012-07-06	2025-03-04	0.859	0.009	Research at Walnut Gulch Experimental Watershed is funded by the USDA-ARS. The Kendall Ameriflux core site is also supported by the Dept. Of Energy Office of Science.<br>This research was a contribution from the Long-Term Agroecosystem Research (LTAR) network. LTAR is supported by the United States Department of Agriculture.
laclaflamme	-71.121453	47.322678	784	EN; WL	0.18	1576	2014-09-10	2025-03-04	0.933	0.687	
laselva2	-84.0071	10.4303	92	EB	25.97	3704	2023-10-12	2025-03-04	0.523	0.5	
laupahoehoe	-155.2912	19.9322	1145	EB	17.57	2189	2016-11-14	2024-05-29	0.157	0	
lethbridge	-112.94025	49.70919	950	GR	5.74	387	2011-12-07	2019-09-13	0.892	0.995	Research at the Lethbridge Grassland Ecosystem site is supported by grants from the Natural Sciences and Engineering Research Council of Canada (RGPIN-2014-05882) to L.B. Flanagan.
lostcreek	-89.9792	46.0827	480	WL	4.11	834	2015-07-28	2025-03-04	0.994	0.861	Support for US-Los is provided from the ChEAS Core Site Cluster by the DOE Office of Science Ameriflux Network Management Project
luckyhills	-110.052023	31.743896	1366	SH	17.06	360	2013-04-30	2025-03-04	0.828	0	Research at Walnut Gulch Experimental Watershed is funded by the USDA-ARS. The Lucky Hills Ameriflux core site is also supported by the Dept. Of Energy

											Office of Science.<br>This research was a contribution from the Long-Term Agroecosystem Research (LTAR) network. LTAR is supported by the United States Department of Agriculture.
macleish	-72.6804	42.4484	251	DB	7.67	1190	2017-02-10	2023-07-14	0.991	0.894	Funded by Smith College
mataflux	-48.6463	-23.061	820	EB	19.12	1279	2023-08-15	2025-03-04	0.357	0.653	
millhaft	-2.29883	52.800796	137	DB	8.94	737	2015-04-11	2025-03-04	0.629	0.37	
millhaft06	-2.2988	52.8008	137	DB	8.94	737	2022-11-10	2025-03-04	0.691	0.484	ECT longterm
millhaft07	-2.2988	52.8008	137	DB	8.94	737	2022-11-10	2025-03-04	0.717	0.461	ECT
millhaft2	-2.2988	52.8008	124	DB; GR	8.94	737	2019-12-03	2025-03-04	0.857	0.656	
millhaftmanormeadow	-2.2975	52.8029	115	DB	8.94	737	2023-03-15	2025-03-04	0.892	0.836	Ecological Continuity Trust
missouriozarks	-92.2	38.7441	219	DB; EN	12.45	1013	2012-03-05	2025-03-04	0.954	0.812	Research at the MOFLUX site is supported by the U.S. Department of Energy, Office of Science, Office of Biological and Environmental Research Program, Climate and Environmental Sciences Division through Oak Ridge National Laboratory's Terrestrial Ecosystem Science – Science Focus Area. ORNL is managed by UT-Battelle, LLC, for the U.S. Department of Energy under contract DE-AC05-00OR22725.<br>This research was a contribution from the Long-Term Agroecosystem Research (LTAR) network. LTAR is supported by the United States Department of Agriculture.
monteblanco	-6.5549	37.0224	20	EB; GR; SH	17.86	500	2017-02-15	2025-03-04	0.79	0.68	ESFRI-LifeWatch Ref. AIC-A-2011-0706 FEDER (FICTS-2014-01-02)
montebondonegrass	11.0458	46.0147	1550	GR	6.31	407	2015-04-30	2025-03-03	0.967	0.802	The research activiteis are supported by Fondazione E. Mach

montenegro	-6.482561	36.992313	12	SH	17.86	507	2016-03-17	2025-03-04	0.905	0.676	This site is maintained by the ICTS Doñana Scientific Reserve (ICTS-2009-39) funded by Spanish Ministry of Science, Innovation and Universities. Phenocam camera was acquired by funding from the Structural Project "Adaptation and improvement of the ICTS e-Infrastructure ICTS-RBD for LifeWatch (FEDER FICTS-2014/01/AIC-A-2011-0706)
montmegmsj	-71.1258	45.4234	599	DB	2.47	1448	2019-09-06	2025-03-04	0.991	0.931	E.W.R. Steacie Memorial Fellowship to Mark Vellend, Natural Sciences and Engineering Research Council of Canada RGPIN and RTI grants to Mark Vellend
morganmonroe2	-86.4131	39.3231	275	DB	11.23	1092	2017-04-04	2025-03-04	0.979	0.843	Research at the Morgan-Monroe Ameriflux site is supported by the US Departement of Energy, Office of Science, Office of Biological and Environmental Research throuth the Ameriflux Management Project administered by Lawrence Berkeley National Lab
nahuku	-155.238383	19.415187	1202	EB	17.02	1933	2015-07-16	2022-05-05	0.939	0.935	Research at the Nahuku site is partially supported by a USDA McIntire-Stennis project at University of Hawaii at Manoa, HI
nevcansnk3a	-114.3095	39.0099	3063	EN	2.64	526	2016-09-19	2025-03-04	0.829	0.414	The Nevada Climate-ecohydrology Assessment Network (NevCAN) is a jointly owned research infrastructure held by three institutions in the Nevada System of Higher Education – the University of Nevada, Reno; the Desert Research Institute, and the University of Nevada, Las Vegas. Site partners include the Long Now Foundation, Great Basin National Park, and the Bureau of Land Management. Please contact NevCAN personnel listed prior to data use or publication.
nevcanspg1a	-	38.9253	1792	SH	8.59	267	2018-	2025-	0.867	0.95	The Nevada Climate-

	114.4082						06-29	03-04			ecohydrology Assessment Network (NevCAN) is a jointly owned research infrastructure held by three institutions in the Nevada System of Higher Education – the University of Nevada, Reno; the Desert Research Institute, and the University of Nevada, Las Vegas. Site partners include the Long Now Foundation, Great Basin National Park, and the Bureau of Land Management. Please contact NevCAN personnel listed prior to data use or publication.
nevcanspg2a	-114.3521	38.8921	2203	EN	6.6	335	2018-06-29	2025-03-04	0.671	0.407	The Nevada Climate-ecohydrology Assessment Network (NevCAN) is a jointly owned research infrastructure held by three institutions in the Nevada System of Higher Education – the University of Nevada, Reno; the Desert Research Institute, and the University of Nevada, Las Vegas. Site partners include the Long Now Foundation, Great Basin National Park, and the Bureau of Land Management. Please contact NevCAN personnel listed prior to data use or publication.
nevcanspg3a	-114.3314	38.8898	2820	EN	2.81	513	2016-09-20	2025-03-04	0.964	0.195	The Nevada Climate-ecohydrology Assessment Network (NevCAN) is a jointly owned research infrastructure held by three institutions in the Nevada System of Higher Education – the University of Nevada, Reno; the Desert Research Institute, and the University of Nevada, Las Vegas. Site partners include the Long Now Foundation, Great Basin National Park, and the Bureau of Land Management. Please contact NevCAN personnel listed prior to data use or publication.
nevcanspg4a	-114.3089	38.9061	3360	EN	2.81	513	2016-09-20	2025-03-04	0.929	0.203	The Nevada Climate-ecohydrology Assessment Network (NevCAN) is a

											jointly owned research infrastructure held by three institutions in the Nevada System of Higher Education – the University of Nevada, Reno; the Desert Research Institute, and the University of Nevada, Las Vegas. Site partners include the Long Now Foundation, Great Basin National Park, and the Bureau of Land Management. Please contact NevCAN personnel listed prior to data use or publication.
niwot3	-105.54697017	40.03286358	3050	EN	1.83	567	2015-07-16	2025-03-04	0.874	0.543	The US-NR1 AmeriFlux site is currently supported by the U.S. DOE, Office of Science through the AmeriFlux Management Project (AMP) at Lawrence Berkeley National Laboratory under Award Number 7094866.
nlloobs	5.7435	52.1666	25	EN	9.43	826	2023-02-14	2025-03-04	0.618	0.596	Velux project, Meteorology and Air Quality dept, Wageningen University, Ruisdael Observatory
northinletsaltmarsh	-79.1957	33.3455	1	WL	17.94	1372	2017-10-12	2025-03-04	0.843	0.537	We thank the Belle W. Baruch Foundation for hosting the site and the North Inlet - Winyah Bay National Estuarine Research Reserve (NI-WB NERR) and the South Carolina Sea Grant Consortium (grant NA18OAR4170091) for support.
ohmcell2	-97.1228	50.1737	231	WL	2.18	522	2023-07-19	2025-03-04	0.8	0.509	This site is supported by Ducks Unlimited Canada with funding from the Government of Canadas Climate Action and Awareness Fund.
ordesa	-0.068269393	42.65105157	1330	DB	5.16	1191	2023-04-18	2025-01-29	0.987	0.828	European Commission – NextGenerationEU (Regulation EU 2020/2094), through CSICs Global Health Platform (PTI Salud Global)
oregonMP	-121.5574	44.4523	1253	EN	6.5	821	2011-06-14	2020-08-20	0.733	0.354	Support for US-Me2 is provided from the Metolius Core Site Cluster by the DOE Office of Science

											Ameriflux Network Management Project
oregonYP	-121.605993	44.32384	977	EN	7.5	537	2011-08-18	2025-03-04	0.856	0.065	Support for US-Me6 is provided from the Metolius Core Site Cluster by the DOE Office of Science Ameriflux Network Management Project
oregonYPbud	-121.606	44.3238	977	EN	7.5	537	2019-05-23	2025-03-04	0.329	0.856	Funding for this AmeriFlux core site was provided by the U.S. Department of Energy's Office of Science
oregonmp1	-121.5574	44.4523	1253	EN	6.5	821	2018-04-30	2020-08-06	0.77	0.353	Funding for this AmeriFlux core site was provided by the U.S. Department of Energy's Office of Science
othshardwood	-68.6655	44.934805	41	UN	6.52	1045	2019-04-08	2025-03-04	0.975	0.772	Funding for this site comes from the NSF Macrosystems Biology Grant 1802726.
othssoftwood	-68.664888	44.934527	41	UN	6.52	1045	2019-04-08	2025-03-04	0.996	0.879	Funding for this site comes from the NSF Macrosystems Biology Grant 1802726.
pace	-78.2739	37.9229	100	DB; EN	13.34	1124	2017-03-09	2024-03-02	0.963	0.835	This site is supported by the Pace Endowment fund to the Department of Environmental Sciences, University of Virginia.
palamanui2	-155.9952	19.7404	240	EB; SH	21.44	2312	2022-02-16	2024-01-23	0.087	0.486	NSF #2026264, NGS #WW-235R-17
piedmontalpasture2	-85.6935	33.8875	238	GR	15.69	1422	2019-11-06	2023-10-16	0.968	0.831	Foundation for Food and Agriculture Research (FFAR), McDonald's Foundation, Shell Foundation, ExxonMobil Foundation
portal	-109.0823	31.9385	1330	SH	16.26	315	2016-05-30	2025-03-04	0.652	0.283	Erica M. Christensen and Ellen K. Bledsoe assisted considerably in the setup and maintenance of the Portal phenocam. This research was supported by the National Science Foundation through grant 1622425 to S.K.M. Ernest.
poudreriver	-104.8141	40.443398	4745	GR	9.49	371	2014-09-01	2016-12-07	0.787	0.377	Research at the poudreriver site is supported by Colorado State University and the AmericaView program (grants G13AC00393, G11AC20461,

											G15AC00056) with phenocam equipment and deployment sponsored by the Department of Interior North Central Climate Science Center.
queens	-76.324	44.565	126	DB; EN	6.4	952	2008-05-26	2025-03-04	0.986	0.82	
quickbird	-109.30387	41.928871	2257	SH	3.67	209	2014-05-23	2015-12-17	0.461	0.394	Research at the quickbird site is supported by the US Geological Survey Wyoming Landscape Conservation Initiative and the Department of Interior North Central Climate Science Center.
riverton	-108.4001	42.9886	1502	GR	7.03	219	2020-11-12	2023-11-02	0.953	0.826	SLAC GROUNDWATER QUALITY SFA, Funded by the DOE Office of Biological and Environmental Research, Climate and Environmental Sciences Division
robinson	-83.1576	37.4671	483	DB	12.3	1181	2017-04-07	2024-05-09	0.962	0.862	University of Kentucky, College of Agriculture, Food, and Environment
robinson2	-83.1576	37.4671	483	DB	12.3	1181	2017-04-07	2024-05-09	0.969	0.879	University of Kentucky, College of Agriculture, Food, and Environment
rosalia	16.3015	47.7027	678	EN	8.07	788	2019-07-05	2025-03-04	0.069	0.196	The camera is operated by the Austrian national weather service, GeoSphere Austria (formerly the Zentralanstalt für Meteorologie und Geodynamik).
russellsage	-91.974322	32.456961	20	DB	18.03	1363	2013-11-20	2025-03-04	0.919	0.791	
sabinar	-6.513228	36.99555	20	EN	17.82	505	2016-06-23	2025-03-04	0.818	0.611	This site is maintained by the ICTS Doñana Scientific Reserve (ICTS-2009-39) funded by Spanish Ministry of Science, Innovation and Universities. Phenocam camera was acquired by funding from the Structural Project "Adaptation and improvement of the ICTS e-Infrastructure ICTS-RBD for LifeWatch (FEDER FICTS-2014/01/AIC-A-

											2011-0706)
sagehen	-120.2398	39.4315	1934	EN	5.77	825	2017-10-05	2025-03-04	0.553	0.563	The PhenoCam for Sagehen Creek Field Station was funded by ETH Zurich.
sagehen2	-120.2407	39.4307	1937	GR	5.77	825	2017-10-13	2025-03-04	0.855	0.647	The PhenoCam for Sagehen Creek Field Station was funded by ETH Zurich.
sagehen3	-120.2405	39.4311	1935	GR	5.77	825	2019-05-10	2025-03-04	0.843	0.673	The PhenoCam for Sagehen Creek Field Station was funded by ETH Zurich.
santaluciapreserve1	-121.8682	36.5265	500	EN	13.16	815	2020-07-10	2025-03-04	0.756	0.673	
sedgwick2	-120.037	34.7012	356	DB; GR	15.61	476	2022-08-09	2025-03-04	0.207	0.203	La Kretz Institute
segaarboretum10	-111.73151	35.16209	2170	GR	7.34	561	2019-05-14	2023-05-24	0.901	0.336	
segaarboretum2	-111.73151	35.16209	2170	GR	7.34	561	2018-11-05	2023-01-19	0.934	0.33	USGS, NAU, NSF
segaarboretum3	-111.73151	35.16209	2170	GR	7.34	561	2019-05-15	2023-01-19	0.857	0.21	
segaarboretum4	-111.73153	35.16214	2170	GR	7.34	561	2018-11-05	2023-01-19	0.99	0.47	USGS, NAU, NSF
segaarboretum6	-111.73151	35.16209	2170	GR	7.34	561	2019-05-14	2023-01-19	0.768	0.123	
segaarboretum9	-111.73158	35.16222	2170	GR	7.34	561	2018-11-05	2023-01-19	0.958	0.4	USGS, NAU, NSF
segaarboretumforest	-111.7359	35.1613	2173	EN; GR	7.34	561	2019-05-29	2025-03-04	0.933	0.458	NSF MRI and FSML funding, Arizona TRIF Funding
segaarboretummeadow	-111.7316	35.1621	2157	EN; GR	7.34	561	2016-08-09	2024-10-20	0.929	0.593	NSF BDI #1126840
segabearsprings	-112.1722	36.3629	2688	EN	4.17	551	2017-11-09	2019-09-14	0.976	0.766	NSF DBI #1126840
segablackpoint	-111.4754	35.6814	1566	GR	13.49	210	2016-01-28	2024-09-28	0.699	0.407	NSF DBI #1126840
segabluechute	-	35.5876	1930	EN;	9.71	415	2017-	2025-	0.95	0.682	NSF DBI #1126840

	111.9716			GR			04-18	03-04			
segabluechute1	-111.97115	35.58759	1931	GR	9.71	415	2019-05-16	2024-06-15	0.777	0.299	USGS, NAU, NSF
segabluechute2	-111.97115	35.58759	1931	GR	9.71	415	2019-05-16	2024-06-15	0.985	0.55	
segabluechute4	-111.9716	35.58759	1931	GR	9.71	415	2019-05-16	2024-06-15	0.535	0.179	
segabluechute6	-111.97115	35.58759	1931	GR	9.71	415	2019-05-16	2024-06-15	0.874	0.556	
segabluechute7	-111.97115	35.58759	1931	GR	9.71	415	2019-05-16	2024-06-15	0.875	0.484	
segabluechute8	-111.97115	35.58759	1931	GR	9.71	415	2019-05-16	2024-06-15	0.86	0.294	
segabradshaw	-111.9285	34.9109	1402	EN	15.61	486	2017-03-15	2025-03-04	0.966	0.509	NSF DBI #1126840
segahartprairie	-111.7324	35.3536	2585	DB; GR	4.63	614	2019-08-02	2025-03-04	0.882	0.679	
segalcr	-111.321	35.7231	1275	GR	14.59	159	2018-07-31	2025-03-04	0.534	0.533	This material is based upon work supported by the National Science Foundation under Grant No. 1126840
segalittlemountain	-112.3634	36.5865	2276	EN; GR	6.95	465	2016-04-01	2022-12-28	0.913	0.751	NSF DBI #1126840
segasoapcreek	-111.716	36.751	1233	SH	13.77	218	2018-07-31	2025-03-04	0.29	0.011	This material is based upon work supported by the National Science Foundation under Grant No. 1126840
segawhitepockets	-112.4082	36.6081	2057	EN; GR	7.95	437	2016-04-01	2025-03-04	0.322	0.442	NSF DBI #1126840
sendadarwinforest	-73.676	-41.8832	30	EB	10.73	2409	2023-01-17	2025-03-04	0.147	0.208	National Agency of Research and Development (ANID, Chile), grants FONDECYT 1211652 and PIA/BASAL FB210006
sendadarwinpeatland	-73.6655	-41.8793	25	WL	10.69	2354	2022-12-16	2025-03-04	0.911	0.844	National Agency of Research and Development (ANID, Chile), grants FONDECYT 1211652 and PIA/BASAL FB210006

sevilletagrass	-106.700186	34.360444	1600	GR	13.02	255	2014-11-05	2025-03-04	0.914	0.482	Support is provided by the Sevilleta Field Station and the University of New Mexico
sevilletanewgrass	-106.6799	34.358	1603	GR	13.02	255	2018-12-05	2025-03-04	0.957	0.542	Ameriflux
sevilletashrub	-106.744469	34.334956	1603	GR; SH	13.02	248	2014-10-29	2025-03-04	0.967	0.13	Support is provided by the Sevilleta Field Station and the University of New Mexico
sevmveblack13redinc	-106.7275	34.3357	1587	GR	13.02	248	2023-02-01	2025-03-04	0.345	0.832	NSF LTER, NSF DEB
sevmveblack14redinc	-106.7275	34.3356	1587	GR	13.02	248	2023-02-01	2025-03-04	0.04	0.918	NSF LTER, NSF DEB
sevmveblack15redamb	-106.7274	34.3356	1587	GR	13.02	248	2023-02-01	2025-03-04	0.111	0.941	NSF LTER, NSF DEB
sevmveblack16ambinc	-106.7276	34.3355	1587	GR	13.02	248	2023-02-02	2025-03-04	0.006	0.846	NSF LTER, NSF DEB
sevmveblack17ambinc	-106.7275	34.3355	1587	GR	13.02	248	2023-02-02	2025-03-04	0.138	0.864	NSF LTER, NSF DEB
sevmveblack18ambamb	-106.7273	34.3355	1587	GR	13.02	248	2023-02-02	2025-03-04	0.08	0.878	NSF LTER, NSF DEB
sevmveblack19ambamb	-106.7272	34.3356	1587	GR	13.02	248	2023-02-02	2025-03-04	0.084	0.888	NSF LTER, NSF DEB
sevmveblack20ambinc	-106.727	34.3356	1587	GR	13.02	248	2023-02-02	2025-03-04	0.089	0.901	NSF LTER, NSF DEB
sevmveblack21ambinc	-106.7269	34.3356	1587	GR	13.02	248	2023-02-02	2025-03-04	0.206	0.943	NSF LTER, NSF DEB
sevmveblack22redamb	-106.7272	34.3355	1587	GR	13.02	248	2023-02-02	2025-03-04	0.262	0.928	NSF LTER, NSF DEB
sevmveblack23redinc	-106.7271	34.3355	1587	GR	13.02	248	2023-02-02	2025-03-04	0.115	0.577	NSF LTER, NSF DEB
sevmveblack24redinc	-106.727	34.3354	1587	GR	13.02	248	2023-02-02	2025-03-04	0.108	0.873	NSF LTER, NSF DEB
sevmveblack25redamb	-106.7275	34.3354	1587	GR	13.02	248	2023-02-02	2025-03-04	0.163	0.963	NSF LTER, NSF DEB
sevmveblack26redinc	-106.7273	34.3354	1587	GR	13.02	248	2023-02-02	2025-03-04	0.343	0.868	NSF LTER, NSF DEB
sevmveblack27redinc	-106.7272	34.3353	1587	GR	13.02	248	2023-02-02	2025-03-04	0.176	0.953	NSF LTER, NSF DEB
sevmveblack28ambinc	-106.7275	34.3353	1587	GR	13.02	248	2023-02-02	2025-03-04	0.071	0.743	NSF LTER, NSF DEB

sevmveblack29ambinc	-106.7274	34.3352	1587	GR	13.02	248	2023-02-02	2025-03-04	0.128	0.933	NSF LTER, NSF DEB
sevmveblack30ambamb	-106.7272	34.3352	1587	GR	13.02	248	2023-02-02	2025-03-04	0.199	0.861	NSF LTER, NSF DEB
sevmveblue10ambinc	-106.6311	34.3341	1646	GR	12.99	261	2022-05-24	2025-03-04	0.021	0.023	NSF LTER, NSF DEB
sevmveblue11ambinc	-106.631	34.3341	1646	GR	12.99	261	2022-05-24	2025-03-04	0.351	0.033	NSF LTER, NSF DEB
sevmveblue12ambamb	-106.6309	34.334	1646	GR	12.99	261	2022-05-24	2025-03-04	0.212	0.007	NSF LTER, NSF DEB
sevmveblue13ambamb	-106.6311	34.334	1646	GR	12.99	261	2022-05-24	2025-03-04	0.287	0	NSF LTER, NSF DEB
sevmveblue14ambinc	-106.6313	34.334	1646	GR	12.99	261	2022-05-24	2025-03-04	0.02	0.034	NSF LTER, NSF DEB
sevmveblue15ambinc	-106.6314	34.334	1646	GR	12.99	261	2022-05-24	2025-03-04	0.252	0.005	NSF LTER, NSF DEB
sevmveblue16redamb	-106.6314	34.3339	1646	GR	12.99	261	2022-05-24	2025-03-04	0.139	0.064	NSF LTER, NSF DEB
sevmveblue17redinc	-106.6313	34.3339	1646	GR	12.99	261	2022-05-24	2025-03-04	0.213	0.066	NSF LTER, NSF DEB
sevmveblue18redinc	-106.6312	34.3338	1646	GR	12.99	261	2022-05-24	2025-03-04	0.4	0.006	NSF LTER, NSF DEB
sevmveblue1ambinc	-106.6307	34.3344	1646	GR	12.99	261	2022-05-24	2025-03-04	0.541	0.914	NSF LTER, NSF DEB
sevmveblue2ambinc	-106.6308	34.3344	1646	GR	12.99	261	2022-05-24	2025-03-04	0.506	0.931	NSF LTER, NSF DEB
sevmveblue3ambamb	-106.6309	34.3345	1646	GR	12.99	261	2022-05-24	2025-03-04	0.71	0.816	NSF LTER, NSF DEB
sevmveblue4redinc	-106.631	34.3344	1646	GR	12.99	261	2022-05-24	2025-03-04	0.634	0.971	NSF LTER, NSF DEB
sevmveblue5redinc	-106.6308	34.3343	1646	GR	12.99	261	2022-05-24	2025-03-04	0.114	0.627	NSF LTER, NSF DEB
sevmveblue6redamb	-106.6307	34.3342	1646	GR	12.99	261	2022-05-24	2025-03-04	0.635	0.896	NSF LTER, NSF DEB
sevmveblue7redinc	-106.6308	34.3341	1646	GR	12.99	261	2022-05-24	2025-03-04	0.416	0.023	NSF LTER, NSF DEB
sevmveblue8redinc	-106.631	34.3342	1646	GR	12.99	261	2022-05-24	2025-03-04	0.337	0.092	NSF LTER, NSF DEB
sevmveblue9redamb	-106.6311	34.3342	1646	GR	12.99	261	2022-05-24	2025-03-04	0.565	0.341	NSF LTER, NSF DEB
sevmvecreo10redamb	-	34.3382	1577	SH	13.02	248	2023-	2025-	0.514	0.063	NSF LTER, NSF DEB

	106.7388						05-21	03-04			
sevmvecreo11redinc	-106.7386	34.3383	1577	SH	13.02	248	2023-05-21	2025-03-04	0.927	0.007	NSF LTER, NSF DEB
sevmvecreo12redinc	-106.7385	34.3382	1577	SH	13.02	248	2023-05-21	2025-03-04	0.541	0.136	NSF LTER, NSF DEB
sevmvecreo19redamb	-106.739	34.3386	1577	SH	13.02	248	2023-05-23	2025-03-04	0.083	0.657	NSF LTER, NSF DEB
sevmvecreo1redinc	-106.7392	34.3382	1577	SH	13.02	248	2023-05-22	2025-03-04	0.001	0.808	NSF LTER, NSF DEB
sevmvecreo20redinc	-106.739	34.3386	1577	SH	13.02	248	2023-05-23	2025-03-04	0.026	0.832	NSF LTER, NSF DEB
sevmvecreo21redinc	-106.739	34.3386	1577	SH	13.02	248	2023-05-23	2025-03-04	0	0.886	NSF LTER, NSF DEB
sevmvecreo22ambinc	-106.739	34.3386	1577	SH	13.02	248	2023-05-23	2025-03-04	0.603	0.077	NSF LTER, NSF DEB
sevmvecreo23ambinc	-106.739	34.3386	1577	SH	13.02	248	2023-05-23	2025-03-04	0.184	0.411	NSF LTER, NSF DEB
sevmvecreo24ambamb	-106.739	34.3386	1577	SH	13.02	248	2023-05-23	2025-03-04	0.152	0.414	NSF LTER, NSF DEB
sevmvecreo2redinc	-106.7391	34.3382	1577	SH	13.02	248	2023-05-22	2025-03-04	0	0.87	NSF LTER, NSF DEB
sevmvecreo3redamb	-106.7389	34.3382	1577	SH	13.02	248	2023-05-22	2025-03-04	0.243	0.657	NSF LTER, NSF DEB
sevmvecreo4ambinc	-106.7392	34.3384	1577	SH	13.02	248	2023-05-22	2025-03-04	0.038	0.826	NSF LTER, NSF DEB
sevmvecreo5ambinc	-106.739	34.3384	1577	SH	13.02	248	2023-05-22	2025-03-04	0.606	0.131	NSF LTER, NSF DEB
sevmvecreo6ambamb	-106.7389	34.3383	1577	SH	13.02	248	2023-05-22	2025-03-04	0.508	0.211	NSF LTER, NSF DEB
sevmvecreo7ambinc	-106.7388	34.3381	1577	SH	13.02	248	2023-05-21	2025-03-04	0.745	0.053	NSF LTER, NSF DEB
sevmvecreo8ambinc	-106.7387	34.3381	1577	SH	13.02	248	2023-05-21	2025-03-04	0.48	0.161	NSF LTER, NSF DEB
sevmvecreo9ambamb	-106.7385	34.3381	1577	SH	13.02	248	2023-05-21	2025-03-04	0.046	0.75	NSF LTER, NSF DEB
sevpjrm12	-106.52621	34.38615	1905	EN	11.93	321	2019-12-10	2023-05-10	0.208	0.157	DEB-1655499, DEB-1702697
sevpjrm13	-106.52791	34.38631	1914	EN	11.93	321	2018-04-14	2023-05-10	0.698	0.803	DEB-1655499, DEB-1702697

sevpjrm15	-106.52838	34.38615	1916	EN	11.93	321	2019-12-10	2021-09-04	0.932	0.333	DEB-1655499, DEB-1702697
sevpjrm16	-106.52897	34.38577	1922	EN	11.93	321	2019-12-10	2021-09-24	0.097	0.022	DEB-1655499, DEB-1702697
sevpjrm17	-106.52837	34.38676	1918	EN	11.93	321	2019-12-10	2021-08-22	0.411	0.023	DEB-1655499, DEB-1702697
shalehillsczo	-77.9041	40.6658	310	DB	9.74	989	2012-04-18	2025-03-04	0.993	0.889	Research at the Penn State Sone Valley Forest is supported by the National Science Foundation EAR 07-25019 (C. Duffy), and EAR 12-39285, EAR 13-31726 (S. Brantley) for the Susquehanna Shale Hills Critical Zone Observatory and the College of Agricultural Sciences, Department of Ecosystem Science and Management.
si04brdo	14.4	46.2872	471	EN	8.58	1543	2022-05-04	2024-05-24	0.701	0.376	GIS
silverplains	147.0871	-42.0912	868	GR	7.79	827	2021-09-01	2025-03-04	0.058	0.97	Research at Silver Plains is supported by the Tasmanian Land Conservancy. Infrastructure supported by the Australian Research Council
silverplainsexpt	147.0875	-42.0908	880	GR	7.79	827	2021-10-18	2024-10-10	0.01	0.936	Research at Silver Plains is supported by the Tasmanian Land Conservancy. Infrastructure supported by the Australian Research Council
slatefloodplain	-107.0282	38.9094	2725	EN; WL	0.42	511	2020-07-31	2025-03-04	0.904	0.78	SLAC GROUNDWATER QUALITY SFA, Funded by the DOE Office of Biological and Environmental Research, Climate and Environmental Sciences Division
snodgrass1	-106.97143762	38.92619636	3023	DB; UN	0.26	500	2021-06-16	2025-03-04	0.972	0.714	Rocky Mountain Biological Laboratorry, USDA Forest Service, DOE Grant # DE-SC0021139
snodgrass2	-106.97143762	38.92619636	3023	UN	0.26	500	2021-06-21	2025-03-04	0.993	0.793	Rocky Mountain Biological Laboratorry, USDA Forest Service, DOE Grant # DE-

											SC0021139
snodgrass3	-106.97811962	38.92839978	3141	DB; UN	0.26	500	2021-06-23	2025-03-04	0.97	0.68	Rocky Mountain Biological Laboratorry, USDA Forest Service, DOE Grant # DE-SC0021139
snodgrass4	-106.97811962	38.92839978	3141	DB; UN	0.26	500	2021-06-23	2025-03-04	0.962	0.719	Rocky Mountain Biological Laboratorry, USDA Forest Service, DOE Grant # DE-SC0021139
snodgrass5	-106.97852093	38.92744789	3152	EN; UN	0.26	500	2021-06-23	2025-03-04	0.971	0.681	Rocky Mountain Biological Laboratorry, USDA Forest Service, DOE Grant # DE-SC0021139
snodgrass6	-106.97852093	38.92744789	3152	EN; UN	0.26	500	2021-06-23	2025-03-04	0.947	0.65	Rocky Mountain Biological Laboratorry, USDA Forest Service, DOE Grant # DE-SC0021139
snodgrass8	-106.98523878	38.93142134	3363	EN; UN	0.26	500	2021-06-12	2025-03-04	0.774	0.443	Rocky Mountain Biological Laboratorry, USDA Forest Service, DOE Grant # DE-SC0021139
somo	10.518	51.765	770	EN; WL	5.12	1500	2023-12-14	2025-03-04	0.775	0.425	University of Göttingen
spruceA0EMI	-93.4541	47.5049	413	EN; SH	3.59	693	2016-03-31	2025-03-04	0.923	0.712	
spruceA0EMT	-93.4541	47.5049	418	SH	3.59	693	2014-10-14	2025-03-04	0.802	0.559	
spruceA0P07	-93.45314	47.50493	413	EN; SH	3.59	693	2016-03-30	2025-03-04	0.916	0.682	
spruceA0P07SH	-93.45314	47.50493	413	SH	3.59	693	2017-11-29	2025-03-04	0.845	0.606	
spruceA0P21SH	-93.45275	47.50625	413	SH	3.59	693	2017-11-29	2025-03-04	0.875	0.623	
spruceT0P06	-93.4535	47.5051	410	DN; EN; SH	3.59	693	2015-08-23	2025-03-04	0.952	0.75	
spruceT0P06SH	-93.4535	47.5051	410	SH	3.59	693	2017-11-29	2025-03-04	0.766	0.504	
spruceT0P19E	-93.4535	47.5065	410	EN; SH	3.59	693	2015-08-23	2025-03-04	0.86	0.613	
spruceT0P19ESH	-93.4535	47.5065	410	SH	3.59	693	2017-11-29	2025-03-04	0.767	0.548	
spruceT2P11E	-93.4527	47.5053	410	DN; EN; SH	3.59	693	2015-08-23	2025-03-04	0.982	0.85	

spruceT2P11ESH	-93.4527	47.5053	410	SH	3.59	693	2017-11-29	2025-03-04	0.729	0.467	
spruceT2P20	-93.4532	47.5064	410	DN; EN; SH	3.59	693	2015-08-23	2025-03-04	0.951	0.749	
spruceT2P20SH	-93.4532	47.5064	410	SH	3.59	693	2017-11-29	2025-03-04	0.768	0.478	
spruceT4P04E	-93.454	47.5052	410	DN; EN; SH	3.59	693	2015-08-23	2025-03-04	0.997	0.872	
spruceT4P04ESH	-93.454	47.5052	410	SH	3.59	693	2017-11-29	2025-03-04	0.686	0.399	
spruceT4P13	-93.453	47.5057	410	DN; EN; SH	3.59	693	2015-08-23	2025-03-04	0.993	0.803	
spruceT4P13SH	-93.453	47.5057	410	SH	3.59	693	2017-11-29	2025-03-04	0.847	0.572	
spruceT6P08	-93.4539	47.5056	410	DN; EN; SH	3.59	693	2015-08-23	2025-03-04	0.986	0.839	
spruceT6P08SH	-93.4539	47.5056	410	SH	3.59	693	2017-11-29	2025-03-04	0.852	0.539	
spruceT6P16E	-93.4532	47.5061	410	DN; EN; SH	3.59	693	2015-08-23	2025-03-04	0.993	0.882	
spruceT6P16ESH	-93.4532	47.5061	410	SH	3.59	693	2017-11-29	2025-03-04	0.922	0.69	
spruceT9P10E	-93.4532	47.5054	410	DN; EN; SH	3.59	693	2015-08-23	2025-03-04	0.994	0.911	
spruceT9P10ESH	-93.4532	47.5054	410	SH	3.59	693	2017-11-29	2025-03-04	0.869	0.585	
spruceT9P17	-93.4527	47.506	410	DN; EN; SH	3.59	693	2015-08-23	2025-03-04	0.991	0.853	
spruceT9P17SH	-93.4527	47.506	410	SH	3.59	693	2017-11-29	2025-03-04	0.979	0.767	
srm	-110.8661	31.8214	1116	GR; SH	18.3	474	2018-07-20	2025-03-04	0.763	0.728	USDA-ARS and AmeriFlux core site funding from DOE<br>This research was a contribution from the Long-Term Agroecosystem Research (LTAR) network. LTAR is supported by the United States Department of Agriculture.

sweetbriar	- 79.08482	37.5734 1	242	DB; EN	13.13	1097	2014-09-09	2024-04-09	0.946	0.785	: We gratefully acknowledge support from USDA NIFA grant # 2017-68002-26612, The Thomas F. and Kate Miller Jeffress Memorial Trust, The Global Change Center at Virginia Tech, and Sweet Briar College.
sweetbriarclearcut	-79.0872	37.5753	242	EN	13.13	1097	2019-01-09	2024-04-10	0.8	0.566	We gratefully acknowledge support from USDA NIFA grant # 2017-68002-26612, The Thomas F. and Kate Miller Jeffress Memorial Trust, The Global Change Center at Virginia Tech, and Sweet Briar College.
swisscanopycrane2e	7.7762	47.4386	538	DB	8.8	1200	2020-01-24	2025-03-04	0.781	0.521	Swiss Federal Office for the Environment FOEN, Swiss National Science Foundation SNSF, University of Basel
swisscanopycrane2n	7.7762	47.4386	538	DB	8.8	1200	2020-01-24	2025-03-04	0.808	0.546	Swiss Federal Office for the Environment FOEN, Swiss National Science Foundation SNSF, University of Basel
swisscanopycrane2s	7.7762	47.4386	538	DB	8.8	1200	2020-01-24	2025-03-04	0.828	0.561	Swiss Federal Office for the Environment FOEN, Swiss National Science Foundation SNSF, University of Basel
swisscanopycrane2w	7.7762	47.4386	538	DB	8.8	1200	2020-01-22	2025-03-04	0.869	0.649	Swiss Federal Office for the Environment FOEN, Swiss National Science Foundation SNSF, University of Basel
sylvania	-89.348	46.242	540	DB; EN	3.76	836	2015-07-30	2025-03-04	0.957	0.862	Support for US-Syv is provided from the ChEAS Core Site Cluster by the DOE Office of Science Ameriflux Network Management Project
tfforest	-70.9505	43.1086	23	DB; EN	8.45	1078	2016-12-08	2025-02-19	0.958	0.861	Research at the Thompson Farm Observatory is supported by NH EPSCoR with support from the National Science Foundation's Research Infrastructure Improvement Award (#EPS 1101245) and by the NH Agricultural Experiment
torrepalacio	-6.4426	36.9905	3	WL	17.96	511	2017-	2025-	0.742	0.943	This site is maintained by

							03-24	03-04			the ICTS Doñana Scientific Reserve (ICTS-2009-39) funded by Spanish Ministry of Science, Innovation and Universities. Phenocam camera was acquired by funding from the Structural Project "Adaptation and improvement of the ICTS e-Infrastructure ICTS-RBD for LifeWatch (FEDER FICTS-2014/01/AIC-A-2011-0706)
turkeypointdbf	-80.5576	42.6353	211	DB	8.36	986	2012-02-10	2025-03-04	0.978	0.94	Research at this site was funded by the Natural Sciences and Engineering Research Council (NSERC) of Canada, Canadian Foundation of Innovation (CFI), Ontario Ministry of Research and Innovation (MRI) and Ontario Ministry of Environment, Conservation and Parks (MECP). Support from Ontario Ministry of Natural Resources and Forestry (OMNRF), St Williams Conservation Reserve Community Council (SWCRCC), Long Point Conservation Authority (LPRCA), Whitside family and McMaster University is also acknowledged.
turkeypointenf39	-80.3573	42.7102	232	EN	8.17	976	2012-01-11	2025-03-04	0.729	0.763	Research at this site was funded by the Natural Sciences and Engineering Research Council (NSERC) of Canada, Canadian Foundation of Innovation (CFI), Ontario Ministry of Research and Innovation (MRI) and Ontario Ministry of Environment, Conservation and Parks (MECP). Support from Ontario Ministry of Natural Resources and Forestry (OMNRF), St Williams Conservation Reserve Community Council (SWCRCC), Long Point Conservation Authority (LPRCA), Whitside family and McMaster University is also acknowledged.
turkeypointenf74	-80.3485	42.70736	216	EN	8.35	970	2012-02-02	2025-03-04	0.765	0.695	Research at this site was funded by the Natural

											Sciences and Engineering Research Council (NSERC) of Canada, Canadian Foundation of Innovation (CFI), Ontario Ministry of Research and Innovation (MRI) and Ontario Ministry of Environment, Conservation and Parks (MECP). Support from Ontario Ministry of Natural Resources and Forestry (OMNRF), St Williams Conservation Reserve Community Council (SWCRCC), Long Point Conservation Authority (LPRCA), Whitside family and McMaster University is also acknowledged.
twodafo	-95.829	29.1658	16	DB	20.44	1200	2019-07-12	2023-05-12	0.989	0.941	
ucnrsanzaborrego	-116.3886	33.2401	214	SH	21.81	187	2019-04-15	2025-02-16	0.953	0.771	University of California Institute for the Study of Ecological & Evolutionary Climate Impact, University of California Natural Reserve System Environmental Information & Technology
ufona	-81.950856	27.3836	25	SH	22.42	1285	2016-03-30	2025-03-04	0.415	0.187	Research at the University of Florida, Range Cattle Research and Education Center phenocam tower is supported by the USDA Long Term Agroecosystem Research (LTAR) Network (https://ltar.ars.usda.gov/), and is made possible by a collaboration between University of Florida cooperative extension and Archbold Biological Station.<br>This research was a contribution from the Long-Term Agroecosystem Research (LTAR) network. LTAR is supported by the United States Department of Agriculture.
usmpj	-106.2543606	34.43845	2126	EN	10.69	395	2013-09-20	2025-03-04	0.911	0.922	
vallesburnedconifer	-106.5321	35.8884	3043	UN	4.03	700	2020-09-21	2025-03-04	0.949	0.943	AmeriFlux
vallesmixedconifer	-	35.9192	2752	EN	4.46	671	2018-	2025-	0.354	0.056	Ameriflux

	106.6142						12-19	03-04			
vallesponderosapine	-106.5964	35.864	2501	EN	5.89	597	2020-09-21	2025-03-04	0.097	0.051	Ameriflux
willowcreek	-90.07912	45.805986	521	DB	4.26	818	2012-04-25	2025-03-04	0.996	0.92	Research at the Willow Creek Ameriflux core site is provided by the Dept. Of Energy Office of Science to the ChEAS Cluster
windriverunderstory	-121.9520833	45.8212833	371	EN	9.05	1966	2013-04-17	2025-03-04	0.769	0.601	Data and logistical support were provided by the Wind River Field Station, a joint scientific venture financed by the Pacific Northwest Research Station and the University of Washington
witnesstree	-72.18956997	42.53556197	354	DB	7.12	1103	2014-11-21	2025-03-04	0.396	0.156	
yakimabasin1	-120.6585	46.9216	892	GR; SH	7.32	477	2022-03-29	2025-03-04	0.486	0.14	This research was supported by the U.S. Department of Energy, Office of Science, Office of Biological and Environmental Research, Environmental System Science (ESS) Program. This contribution originates from the River Corridor Scientific Focus Area project at Pacific Northwest National Laboratory (PNNL). PNNL is a multiprogram national laboratory operated for DOE by Battelle Memorial Institute under contract DE-AC06-76RL01830.
yakimabasin2	-120.6241	46.9045	832	GR	7.85	377	2022-03-29	2024-12-17	0.837	0.553	This research was supported by the U.S. Department of Energy, Office of Science, Office of Biological and Environmental Research, Environmental System Science (ESS) Program. This contribution originates from the River Corridor Scientific Focus Area project at Pacific Northwest National Laboratory (PNNL). PNNL is a multiprogram national laboratory operated for DOE by Battelle Memorial Institute under contract DE-AC06-76RL01830.
zarnekow1	12.8891	53.876	10	WL	8.33	594	2021-	2025-	0.488	0.314	TERENO

							04-14	03-04			

Supplementary Table 3: FLUXNET sites and LSP evaluation results. Site information and evaluation results for all FLUXNET daily GPP datasets used for comparison with SIF and NIR_V LSP datasets. International Geosphere-Biosphere Programme (IGBP) land cover classes include: ENF = evergreen needleleaf forest; EBF = evergreen broadleaf forest; DBF = deciduous broadleaf forest; MF = mixed forest; CSH = closed shrublands; OSH = open shrublands; WSA = woody savanna; SAV = savanna; GRA = grasslands; WET = permanent wetlands. MAT = mean annual temperature, in degrees Celsius. MAP = mean annual precipitation, in mm. R^2 values indicate the percent variation in the phenocycle fitted to an LSP (NIR_V or SIF) dataset that is explained by the variation in a GPP dataset's fitted annual cycle.

FLUXNET ID	Site name	Longitude	Latitude	IGBP land cover	MAT (℃)	MAP (mm)	R^2, NIR_v	R^2, SIF
AR-SLu	San Luis	-66.4598	-33.4648	MF		400	0.888	0.971
AR-Vir	Virasoro	-56.1886	-28.2395	ENF			0.412	0.514
AT-Neu	Neustift	11.3175	47.1167	GRA	6.5	852	0.834	0.967
AU-ASM	Alice Springs	133.249	-22.283	SAV			0.776	0.793
AU-Ade	Adelaide River	131.1178	-13.0769	WSA			0.738	0.876
AU-Cpr	Calperum	140.5891	-34.0021	SAV			0.06	0.953
AU-Cum	Cumberland Plain	150.7236	-33.6152	EBF			0.934	0.518
AU-DaP	Daly River Savanna	131.3181	-14.0633	GRA	27.25	983.78	0.922	0.99
AU-DaS	Daly River Cleared	131.3881	-14.1593	SAV	27.22	975.82	0.99	0.929
AU-Dry	Dry River	132.3706	-15.2588	SAV			0.955	0.777
AU-Emr	Emerald	148.4746	-23.8587	GRA			0.704	0.508
AU-Fog	Fogg Dam	131.3072	-12.5452	WET	27.02	1323.69	0.889	0.666
AU-GWW	Great Western Woodlands, Western Australia, Australia	120.6541	-30.1913	SAV			0.012	0.397
AU-Gin	Gingin	115.7138	-31.3764	WSA			0.078	0.704
AU-How	Howard Springs	131.1523	-12.4943	WSA	27.01	1449.35	0.994	0.965
AU-Lox	Loxton	140.6551	-34.4704	DBF			0.123	0.474
AU-RDF	Red Dirt Melon Farm, Northern Territory	132.4776	-14.5636	WSA			0.431	0.64
AU-Rig	Riggs Creek	145.5759	-36.6499	GRA			0.755	0.575
AU-Rob	Robson Creek, Queensland,	145.6301	-17.1175	EBF			0.007	0.006

	Australia							
AU-Stp	Sturt Plains	133.3502	-17.1507	GRA			0.958	0.806
AU-TTE	Ti Tree East	133.64	-22.287	GRA			0.543	0.877
AU-Tum	Tumbarumba	148.1517	-35.6566	EBF	10.72	1159.01	0.105	0.73
AU-Wac	Wallaby Creek	145.1878	-37.4259	EBF	12.76	1105.59	0.216	0.258
AU-Whr	Whroo	145.0294	-36.6732	EBF			0.601	0.256
AU-Wom	Wombat	144.0944	-37.4222	EBF			0.136	0.595
AU-Ync	Jaxa	146.2907	-34.9893	GRA			0.954	0.963
BE-Bra	Brasschaat	4.5198	51.3076	MF	9.8	750	0.957	0.946
BE-Vie	Vielsalm	5.9981	50.3049	MF	7.8	1062	0.936	0.945
BR-Sa1	Santarem-Km67-Primary Forest	-54.9589	-2.8567	EBF	26.13	2074.79	0.587	0.676
BR-Sa3	Santarem-Km83-Logged Forest	-54.9714	-3.018	EBF	26.12	2043.77	0.777	0.844
CA-Gro	Ontario - Groundhog River, Boreal Mixedwood Forest	-82.1556	48.2167	MF	1.3	831	0.977	0.965
CA-Man	Manitoba - Northern Old Black Spruce (former BOREAS Northern Study Area)	-98.4808	55.8796	ENF	-3.2	520	0.971	0.924
CA-NS1	UCI-1850 burn site	-98.4839	55.8792	ENF	-2.89	500.29	0.974	0.905
CA-NS2	UCI-1930 burn site	-98.5247	55.9058	ENF	-2.88	499.82	0.977	0.92
CA-NS3	UCI-1964 burn site	-98.3822	55.9117	ENF	-2.87	502.22	0.958	0.943
CA-NS4	UCI-1964 burn site wet	-98.3806	55.9144	ENF	-2.87	502.22	0.947	0.938
CA-NS5	UCI-1981 burn site	-98.485	55.8631	ENF	-2.86	500.34	0.97	0.932
CA-NS6	UCI-1989 burn site	-98.9644	55.9167	OSH	-3.08	495.37	0.963	0.902
CA-NS7	UCI-1998 burn site	-99.9483	56.6358	OSH	-3.52	483.27	0.943	0.895
CA-Oas	Saskatchewan - Western Boreal, Mature Aspen	-106.1978	53.6289	DBF	0.34	428.53	0.967	0.912
CA-Obs	Saskatchewan - Western Boreal, Mature Black Spruce	-105.1178	53.9872	ENF	0.79	405.6	0.972	0.922
CA-Qfo	Quebec - Eastern Boreal, Mature Black Spruce	-74.3421	49.6925	ENF	-0.36	962.32	0.957	0.929
CA-SF1	Saskatchewan - Western Boreal, forest burned in 1977	-105.8176	54.485	ENF	0.4	470	0.837	0.94

CA-SF2	Saskatchewan - Western Boreal, forest burned in 1989	-105.8775	54.2539	ENF	0.4	470	0.934	0.781
CA-SF3	Saskatchewan - Western Boreal, forest burned in 1998	-106.0053	54.0916	OSH	0.4	470	0.926	0.949
CA-TP1	Ontario - Turkey Point 2002 Plantation White Pine	-80.5595	42.6609	ENF	8	1036	0.903	0.884
CA-TP2	Ontario - Turkey Point 1989 Plantation White Pine	-80.4588	42.7744	ENF	8	1036	0.925	0.848
CA-TP3	Ontario - Turkey Point 1974 Plantation White Pine	-80.3483	42.7068	ENF	8	1036	0.923	0.943
CA-TP4	Ontario - Turkey Point 1939 Plantation White Pine	-80.3574	42.7102	ENF	8	1036	0.858	0.945
CA-TPD	Ontario - Turkey Point Mature Deciduous	-80.5577	42.6353	DBF	8	1036	0.892	0.987
CG-Tch	Tchizalamou	11.6564	-4.2892	SAV	25.7	1150	0.981	0.992
CH-Cha	Chamau	8.4104	47.2102	GRA	9.5	1136	0.93	0.936
CH-Dav	Davos	9.8559	46.8153	ENF	2.8	1062	0.74	0.776
CH-Fru	Früebüel	8.5378	47.1158	GRA	7.2	1651	0.937	0.986
CH-Lae	Laegern	8.3644	47.4783	MF	8.3	1100	0.916	0.864
CH-Oe1	Oensingen grassland	7.7319	47.2858	GRA	9	1100	0.911	0.89
CN-Cha	Changbaishan	128.0958	42.4025	MF	2.16	663.59	0.959	0.839
CN-Cng	Changling	123.5092	44.5934	GRA			0.846	0.998
CN-Dan	Dangxiong	91.0664	30.4978	GRA	-1.54	246.88	0.902	0.967
CN-Din	Dinghushan	112.5361	23.1733	EBF	19.64	1618.1	0.736	0.515
CN-Du2	Duolun_grassland (D01)	116.2836	42.0467	GRA	2.01	318.96	0.83	0.992
CN-Du3	Duolun Degraded Meadow	116.2809	42.0551	GRA			0.889	0.746
CN-Ha2	Haibei Shrubland	101.3269	37.6086	WET			0.94	0.981
CN-HaM	Haibei Alpine Tibet site	101.18	37.37	GRA			0.98	0.827
CN-Qia	Qianyanzhou	115.0581	26.7414	ENF	18.95	1466.75	0.795	0.959
CN-Sw2	Siziwang Grazed (SZWG)	111.8971	41.7902	GRA			0.164	0.489
CZ-BK1	Bily Kriz forest	18.5369	49.5021	ENF	6.7	1316	0.948	0.858
CZ-BK2	Bily Kriz grassland	18.5429	49.4944	GRA	6.7	1316	0.967	0.936
CZ-wet	Trebon (CZECHWET)	14.7704	49.0247	WET	7.7	604	0.916	0.959

DE-Akm	Anklam	13.6834	53.8662	WET	8.7	558	0.549	0.618
DE-Gri	Grillenburg	13.5126	50.95	GRA	7.8	901	0.893	0.82
DE-Hai	Hainich	10.4522	51.0792	DBF	8.3	720	0.915	0.801
DE-Lkb	Lackenberg	13.3047	49.0996	ENF	4	1599	0.986	0.915
DE-Lnf	Leinefelde	10.3678	51.3282	DBF	6.96	894.6	0.904	0.752
DE-Obe	Oberbärenburg	13.7213	50.7867	ENF	5.5	996	0.933	0.889
DE-RuR	Rollesbroich	6.3041	50.6219	GRA	7.7	1033	0.914	0.915
DE-SfN	Schechenfilz Nord	11.3275	47.8064	WET	8.6	1127	0.805	0.659
DE-Spw	Spreewald	14.0337	51.8922	WET	8.7	558	0.934	0.957
DE-Tha	Tharandt	13.5651	50.9626	ENF	8.2	843	0.655	0.563
DE-Zrk	Zarnekow	12.889	53.8759	WET	8.7	584	0.909	0.854
DK-Eng	Enghave	12.1918	55.6905	GRA	8	613	0.517	0.701
DK-Sor	Soroe	11.6446	55.4859	DBF	8.2	660	0.851	0.795
ES-Amo	Amoladeras	-2.2523	36.8336	OSH		0	0.788	0.614
ES-LJu	Llano de los Juanes	-2.7521	36.9266	OSH	16	400	0.876	0.904
ES-LgS	Laguna Seca	-2.9658	37.0979	OSH			0.565	0.866
ES-Ln2	Lanjaron-Salvage logging	-3.4758	36.9695	OSH			0.457	0.579
FR-Fon	Fontainebleau-Barbeau	2.7801	48.4764	DBF	10.2	720	0.934	0.831
FR-LBr	Le Bray	-0.7693	44.7171	ENF	13.6	900	0.82	0.94
FR-Pue	Puechabon	3.5957	43.7413	EBF	13.5	883	0.531	0.812
GF-Guy	Guyaflux (French Guiana)	-52.9249	5.2788	EBF	25.7	3041	0.2	0.044
GH-Ank	Ankasa	-2.6942	5.2685	EBF	26	1900	0.001	0.345
IT-CA1	Castel d'Asso1	12.0266	42.3804	DBF	14	766	0.002	0.258
IT-CA3	Castel d'Asso3	12.0222	42.38	DBF	14	766	0.011	0.205
IT-Col	Collelongo	13.5881	41.8494	DBF	6.3	1180	0.921	0.823
IT-Cp2	Castelporziano2	12.3573	41.7043	EBF	15.2	805	0.783	0.778
IT-Cpz	Castelporziano	12.3761	41.7052	EBF	15.6	780	0.7	0.9
IT-Isp	Ispra ABC-IS	8.6336	45.8126	DBF	12.2	1300	0.836	0.914
IT-La2	Lavarone2	11.2853	45.9542	ENF	7.2	1150	0.955	0.767

IT-Lav	Lavarone	11.2813	45.9562	ENF	7.8	1291	0.86	0.821
IT-MBo	Monte Bondone	11.0458	46.0147	GRA	5.1	1214	0.972	0.971
IT-Noe	Arca di Noe - Le Prigionette	8.1517	40.6062	CSH	15.9	588	0.1	0.393
IT-PT1	Parco Ticino forest	9.061	45.2009	DBF	12.7	984	0.686	0.939
IT-Ren	Renon	11.4337	46.5869	ENF	4.7	809.3	0.906	0.946
IT-Ro1	Roccarespampani 1	11.93	42.4081	DBF	15.15	876.2	0.159	0.258
IT-Ro2	Roccarespampani 2	11.9209	42.3903	DBF	15.15	876.2	0.011	0.185
IT-SR2	San Rossore 2	10.2909	43.732	ENF	14.2	920	0.634	0.808
IT-SRo	San Rossore	10.2844	43.7279	ENF	14.2	920	0.877	0.978
IT-Tor	Torgnon	7.5781	45.8444	GRA	2.9	920	0.885	0.716
JP-MBF	Moshiri Birch Forest Site	142.3186	44.3869	DBF			0.969	0.957
JP-SMF	Seto Mixed Forest Site	137.0788	35.2617	MF			0.991	0.925
MY-PSO	Pasoh Forest Reserve (PSO)	102.3062	2.973	EBF			0.037	0.066
NL-Hor	Horstermeer	5.0713	52.2403	GRA	10	800	0.852	0.871
NL-Loo	Loobos	5.7436	52.1666	ENF	9.8	786	0.931	0.945
PA-SPn	Sardinilla Plantation	-79.6346	9.3181	DBF	26.5	2350	0.95	0.857
PA-SPs	Sardinilla-Pasture	-79.6314	9.3138	GRA	26.5	2350	0.892	0.39
RU-Fyo	Fyodorovskoye	32.9221	56.4615	ENF	3.9	711	0.984	0.928
RU-Ha1	Hakasia steppe	90.0022	54.7252	GRA	-0.07	591.87	0.815	0.927
SD-Dem	Demokeya	30.4783	13.2829	SAV	26	320	0.804	0.872
SN-Dhr	Dahra	-15.4322	15.4028	SAV	29	404	0.873	0.95
US-AR1	ARM USDA UNL OSU Woodward Switchgrass 1	-99.42	36.4267	GRA			0.711	0.885
US-AR2	ARM USDA UNL OSU Woodward Switchgrass 2	-99.5975	36.6358	GRA			0.535	0.731
US-ARb	ARM Southern Great Plains burn site- Lamont	-98.0402	35.5497	GRA			0.759	0.783
US-ARc	ARM Southern Great Plains control site- Lamont	-98.04	35.5465	GRA			0.731	0.779
US-Blo	Blodgett Forest	-120.6328	38.8953	ENF	11.09	1226	0.823	0.851
US-Cop	Corral Pocket	-109.39	38.09	GRA			0.415	0.277
US-GBT	GLEES Brooklyn Tower	-106.2397	41.3658	ENF	0.8	1200	0.782	0.933

US-GLE	GLEES	-106.2399	41.3665	ENF	0.8	1200	0.808	0.88
US-Goo	Goodwin Creek	-89.8735	34.2547	GRA	15.89	1425.77	0.977	0.933
US-Ha1	Harvard Forest EMS Tower (HFR1)	-72.1715	42.5378	DBF	6.62	1071	0.98	0.938
US-KS1	Kennedy Space Center (slash pine)	-80.6709	28.4583	ENF	21.9	1266	0.277	0.724
US-KS2	Kennedy Space Center (scrub oak)	-80.6715	28.6086	CSH	21.66	1294	0.979	0.938
US-LWW	Little Washita Watershed	-97.9789	34.9604	GRA	16.09	805.12	0.784	0.869
US-Los	Lost Creek	-89.9792	46.0827	WET	4.08	828	0.919	0.938
US-MMS	Morgan Monroe State Forest	-86.4131	39.3232	DBF	10.85	1032	0.975	0.928
US-Me1	Metolius - Eyerly burn	-121.5	44.5794	ENF	7.88	704.61	0.789	0.859
US-Me2	Metolius mature ponderosa pine	-121.5574	44.4523	ENF	6.28	523	0.629	0.924
US-Me3	Metolius-second young aged pine	-121.6078	44.3154	ENF	7.07	719	0.131	0.928
US-Me4	Metolius-old aged ponderosa pine	-121.6224	44.4992	ENF	7.61	1038.82	0.578	0.488
US-Me5	Metolius-first young aged pine	-121.5668	44.4372	ENF	6.47	590.81	0.013	0.963
US-Me6	Metolius Young Pine Burn	-121.6078	44.3233	ENF	7.59	494	0.14	0.952
US-Myb	Mayberry Wetland	-121.765	38.0499	WET	15.9	338	0.373	0.426
US-NR1	Niwot Ridge Forest (LTER NWT1)	-105.5464	40.0329	ENF	1.5	800	0.898	0.798
US-Oho	Oak Openings	-83.8438	41.5545	DBF	10.1	849	0.964	0.968
US-PFa	Park Falls/WLEF	-90.2723	45.9459	MF	4.33	823	0.98	0.939
US-SRC	Santa Rita Creosote	-110.8395	31.9083	OSH	22	330	0.515	0.78
US-SRG	Santa Rita Grassland	-110.8277	31.7894	GRA	17	420	0.866	0.954
US-SRM	Santa Rita Mesquite	-110.8661	31.8214	WSA	17.92	380	0.836	0.984
US-Sta	Saratoga	-106.8024	41.3966	OSH	5.1		0.571	0.976
US-Syv	Sylvania Wilderness Area	-89.3477	46.242	MF	3.81	826	0.942	0.923
US-Ton	Tonzi Ranch	-120.966	38.4309	WSA	15.8	559	0.606	0.378
US-Tw1	Twitchell Wetland West Pond	-121.6469	38.1074	WET	15.5	421	0.879	0.592
US-Tw4	Twitchell East End Wetland	-121.6413	38.1027	WET	15.6	421	0.657	0.131

US-UMB	Univ. of Mich. Biological Station	-84.7138	45.5598	DBF	5.83	803	0.926	0.936
US-UMd	UMBS Disturbance	-84.6975	45.5625	DBF	5.83	803	0.918	0.924
US-Var	Vaira Ranch- Ione	-120.9508	38.4133	GRA	15.8	559	0.961	0.981
US-WCr	Willow Creek	-90.0799	45.8059	DBF	4.02	787	0.97	0.934
US-WPT	Winous Point North Marsh	-82.9962	41.4646	WET	10.1	849	0.882	0.939
US-Whs	Walnut Gulch Lucky Hills Shrub	-110.0522	31.7438	OSH	17.6	320	0.848	0.174
US-Wi0	Young red pine (YRP)	-91.0814	46.6188	ENF			0.539	0.904
US-Wi1	Intermediate hardwood (IHW)	-91.2329	46.7305	DBF			0.855	0.605
US-Wi2	Intermediate red pine (IRP)	-91.1528	46.6869	ENF			0.592	0.517
US-Wi3	Mature hardwood (MHW)	-91.0987	46.6347	DBF			0.974	0.873
US-Wi4	Mature red pine (MRP)	-91.1663	46.7393	ENF			0.866	0.774
US-Wi5	Mixed young jack pine (MYJP)	-91.0858	46.6531	ENF			0.803	0.866
US-Wi6	Pine barrens #1 (PB1)	-91.2982	46.6249	OSH			0.893	0.756
US-Wi7	Red pine clearcut (RPCC)	-91.0693	46.6491	OSH			0.952	0.937
US-Wi8	Young hardwood clearcut (YHW)	-91.2524	46.7223	DBF			0.904	0.624
US-Wi9	Young Jack pine (YJP)	-91.0746	46.7385	ENF			0.784	0.885
US-Wkg	Walnut Gulch Kendall Grasslands	-109.9419	31.7365	GRA	15.64	407	0.915	0.152
ZM-Mon	Mongu	23.2525	-15.4391	DBF	25	945	0.983	0.971

Supplementary Table 4: iNaturalist flowering phenology significant MMRR results. Results of analysis of allochrony by allopatry in iNaturalist flowering observations. Rows report the multiple matrix regression with randomization (MMRR) results for all taxa with a significant correlation, after false discovery rate control, between pairwise differences in the NIR_V phenocycles at all iNaturalist flowering observation locations and pairwise differences between those locations' observation dates. Columns give the taxon scientific name (Taxon) and iNaturalist taxon ID (iNat ID); the estimated coefficients and P values for the intercept (int.) and the pairwise geographic distance (geo.), pairwise bioclimatic environmental distance (env.), and pairwise LSP distance (LSP) covariates; and the MMRR model's overall F-statistic (F), its P value (F P) and its R^2. The table of all results, including insignificant taxa, is included in the archived data for this study.

Taxon	iNat ID	n	int.	int. P	geo.	geo. P	env.	env. P	LSP	LSP P	F	F P	R^2
Datura wrightii	58331	569	0.0000	0.2763	-0.1115	0.0001	0.0618	0.0037	0.0711	0.0001	554.5443	0.0001	0.0102
Leonotis ocymifolia	403274	121	0.0000	0.6493	0.1190	0.0211	-0.0554	0.2030	0.2149	0.0001	148.4006	0.0001	0.0578
Satyrium parviflorum	556782	81	0.0000	0.0448	-0.0709	0.0950	0.4811	0.0001	0.3366	0.0001	1007.0214	0.0001	0.4828
Pelargonium sidoides	568517	81	0.0000	0.4970	-0.0688	0.2891	0.0701	0.1856	0.3952	0.0001	198.8545	0.0001	0.1557
Pelargonium englerianum	591582	79	0.0000	0.4633	-0.0188	0.6839	0.1229	0.0043	0.1968	0.0001	65.4647	0.0001	0.0600
Eriogonum deflexum	76981	68	0.0000	0.9637	0.1500	0.0326	-0.1170	0.1217	0.2409	0.0001	63.5397	0.0001	0.0773
Menodora scabra	77988	54	0.0000	0.3621	-0.0084	0.8330	0.0034	0.9403	0.2867	0.0001	42.2645	0.0001	0.0816
Turnera diffusa	170054	39	0.0000	0.6243	-0.3830	0.0003	0.2764	0.0004	0.3641	0.0001	26.3973	0.0002	0.0970
Voyria tenella	291753	37	0.0000	0.0964	-0.1004	0.1311	0.0430	0.4661	0.3304	0.0001	26.5473	0.0001	0.1074
Lycianthes lycioides	526642	27	0.0000	0.1425	-0.1577	0.2887	0.1175	0.2251	0.6093	0.0001	51.0419	0.0001	0.3062
Ipomoea bahiensis	906939	24	0.0000	0.4233	-0.2043	0.0319	0.1438	0.0929	0.5020	0.0001	28.3360	0.0001	0.2381
Raoulia hookeri	405638	22	0.0000	0.0067	0.0087	0.9376	0.0918	0.4669	0.6949	0.0001	99.0732	0.0001	0.5670
Scleranthus annuus	61935	21	0.0000	0.0462	0.4258	0.0005	-0.1012	0.4794	0.3472	0.0001	23.9916	0.0001	0.2589
Allionia incarnata	75334	282	0.0000	0.8255	-0.0257	0.0571	0.0642	0.0001	0.0450	0.0001	83.9706	0.0001	0.0063
Malacothamnus enigmaticus	949749	35	0.0000	0.9349	-0.0943	0.2210	0.0500	0.4655	0.3541	0.0001	33.7776	0.0001	0.1464
Marsypianthes chamaedrys	118916	35	0.0000	0.3227	0.1704	0.1743	-0.1782	0.1015	0.4427	0.0001	48.2955	0.0002	0.1969
Epidendrum fimbriatum	202324	46	0.0000	0.4549	-0.0219	0.4247	-0.0939	0.0149	0.1678	0.0003	5.1699	0.0030	0.0148

Tarchonanthus littoralis	595129	38	0.0000	0.9652	-0.0651	0.5239	-0.0199	0.8563	0.4343	0.0003	47.9152	0.0004	0.1706
Monochaetum myrtoideum	538474	54	0.0000	0.0117	0.0723	0.2933	0.0796	0.1920	0.3178	0.0003	72.9315	0.0001	0.1329
Guzmania monostachia	163447	45	0.0000	0.1824	0.1032	0.0254	0.0551	0.1465	0.2135	0.0003	29.2798	0.0001	0.0818
Wedelia hispida	244815	227	0.0000	0.9896	-0.0091	0.8096	-0.0193	0.5742	0.0905	0.0004	47.5374	0.0014	0.0055
Pelargonium abrotanifolium	574124	44	0.0000	0.7336	0.0491	0.5903	0.0016	0.9816	0.3120	0.0004	43.1319	0.0002	0.1208
Gentianella cerastioides	339485	59	0.0000	0.6103	-0.1155	0.0065	-0.0330	0.2621	0.1486	0.0005	7.9840	0.0065	0.0138
Mimosa strigillosa	165400	65	0.0000	0.3999	0.0551	0.1907	0.0376	0.4422	0.2062	0.0005	44.2918	0.0001	0.0602
Senecio elegans	79044	12	0.0000	0.0522	-0.1205	0.1792	-0.0425	0.7352	0.8314	0.0007	32.3547	0.0012	0.6102
Physalis peruviana	51988	12	0.0000	0.5660	0.7740	0.0037	-0.0065	0.9621	-0.7197	0.0008	6.5659	0.0122	0.2411
Weinmannia tomentosa	534976	40	0.0000	0.5375	-0.0755	0.0757	0.0970	0.0313	0.1950	0.0011	12.7760	0.0023	0.0471
Melochia pyramidata	165294	44	0.0000	0.4709	0.1074	0.2013	-0.1056	0.2114	0.2197	0.0011	14.8474	0.0023	0.0451
Acourtia wrightii	157997	86	0.0000	0.8063	0.3207	0.0001	-0.1013	0.0335	0.1942	0.0014	239.2555	0.0001	0.1643
Vallea stipularis	465262	41	0.0000	0.5985	-0.0699	0.2059	-0.0326	0.4461	0.2264	0.0015	8.6012	0.0053	0.0307
Ledebouria revoluta	490656	27	0.0000	0.1046	-0.0202	0.8723	0.0491	0.6899	0.3563	0.0015	17.4123	0.0013	0.1308
Xyris smalliana	170375	41	0.0000	0.9593	-0.3862	0.0011	0.6674	0.0001	0.1260	0.0019	48.9961	0.0001	0.1526
Erythranthe suksdorfii	533374	31	0.0000	0.5299	-0.0816	0.4051	0.3464	0.0151	0.3993	0.0023	98.7540	0.0001	0.3912
Daboecia cantabrica	371651	48	0.0000	0.9056	-0.0263	0.6806	0.0492	0.4087	0.2316	0.0025	25.3966	0.0019	0.0635
Senecio flaccidus	58036	133	0.0000	0.1337	0.0878	0.0007	0.0189	0.3861	0.0671	0.0026	62.0981	0.0001	0.0208
Tritoniopsis antholyza	570469	53	0.0000	0.1665	0.1852	0.0066	0.0000	0.9997	0.1675	0.0027	39.3293	0.0001	0.0791
Zaluzianskya capensis	119500	17	0.0000	0.4895	-0.1193	0.5281	0.0130	0.9545	0.4628	0.0029	10.1790	0.0203	0.1879
Iris adriatica	902039	26	0.0000	0.0032	0.0000	0.6738	0.5000	0.0032	0.5000	0.0032	2.6961E+32	0.0032	1.0000
Chloropyron maritimum	76296	10	0.0000	0.0275	0.1578	0.3288	-0.1859	0.3758	0.6609	0.0033	11.7860	0.0033	0.4631
Salvia ballotiflora	168376	271	0.0000	0.0503	-0.0860	0.0001	0.0332	0.1233	0.0474	0.0035	37.4786	0.0015	0.0031
Columnea strigosa	545663	38	0.0000	0.7190	-0.0582	0.3091	-0.0128	0.8002	0.2445	0.0040	11.3195	0.0093	0.0463
Celosia argentea	160259	55	0.0000	0.8192	0.0019	0.9224	-0.0244	0.3655	0.0665	0.0041	1.5604	0.0392	0.0032

Lecocarpus darwinii	327248	13	0.0000	0.6577	-0.1322	0.4175	-0.1175	0.4105	0.4444	0.0041	4.5517	0.0404	0.1558
