# Supplementary material for: Global phenology maps reveal the drivers and effects of seasonal asynchrony
Source: Nature. 2025 Aug 27;645(8079):133–40. doi: 10.1038/s41586-025-09410-3 (PMC12408380; doi:10.1038/s41586-025-09410-3)
Supplement: Supplementary file 2 — Reporting Summary [file 41586_2025_9410_MOESM2_ESM.pdf]

nature portfolio

Corresponding author(s): Drew E. Terasaki Hart

Last updated by author(s): June 16, 2025

Reporting Summary

Nature Portfolio wishes to improve the reproducibility of the work that we publish. This form provides structure for consistency and transparency in reporting. For further information on Nature Portfolio policies, see our Editorial Policies and the Editorial Policy Checklist.

Statistics

For all statistical analyses, confirm that the following items are present in the figure legend, table legend, main text, or Methods section.

n/a	Confirmed	
☐	☒	The exact sample size (*n*) for each experimental group/condition, given as a discrete number and unit of measurement
☐	☒	A statement on whether measurements were taken from distinct samples or whether the same sample was measured repeatedly
☐	☒	The statistical test(s) used AND whether they are one- or two-sided<br>*Only common tests should be described solely by name; describe more complex techniques in the Methods section.*
☐	☒	A description of all covariates tested
☐	☒	A description of any assumptions or corrections, such as tests of normality and adjustment for multiple comparisons
☐	☒	A full description of the statistical parameters including central tendency (e.g. means) or other basic estimates (e.g. regression coefficient) AND variation (e.g. standard deviation) or associated estimates of uncertainty (e.g. confidence intervals)
☐	☒	For null hypothesis testing, the test statistic (e.g. *F*, *t*, *r*) with confidence intervals, effect sizes, degrees of freedom and *P* value noted<br>*Give P values as exact values whenever suitable.*
☒	☐	For Bayesian analysis, information on the choice of priors and Markov chain Monte Carlo settings
☒	☐	For hierarchical and complex designs, identification of the appropriate level for tests and full reporting of outcomes
☒	☐	Estimates of effect sizes (e.g. Cohen's *d*, Pearson's *r*), indicating how they were calculated

Our web collection on statistics for biologists contains articles on many of the points above.

Software and code

Policy information about availability of computer code

Data collection	Custom Bash (version 4) and Python (3.7) scripts were used to facilitate download of some SIF data and upload of that data to Google Earth Engine, and custom R (4.0) and Python (3.9) scripts were used to download PhenoCam and iNaturalist data.

Data analysis

Data analysis used custom code written in a variety of languages and using a variety of packages on three different computing environments: 1. Google Earth Engine (via version >=0.1.404 of the browser-hosted Javascript API); 2. a local computing environment, including Pop!_OS (22.04 LTS), Bash (5.1.6), GDAL (3.3.1), Python (3.9, with numpy (1.22.4), rasterio (1.2.10), xarray (2022.3.0), rioxarray (0.15.0), pandas (2.2.1), shapely (2.0.3), geopandas (0.14.3), zipfile36 (0.1.3), tensorflow (2.4.1), pyproj (3.6.1), cartopy (0.20.2), geopy (1.13.0), scipy (1.13.0), sklearn (1.0.2), alphashape (1.3.1), rasterstats (0.16.0), statsmodels (0.13.2), seaborn (0.11.2), fuzzywuzzy (0.18.0), Bio (1.79), pyinaturalist (0.19.0), json (2.0.9), nlmpy (no version), matplotlib (3.7.0), h3 (3.7.4), decartes (no version), contextily (1.2.0), xyzservices (2022.4.0), palettable (3.3.0), cmocean (2.0), cmcrameri (1.8), colormap (1.0.4), imageio (2.19.0), and cv2 (4.9.0)), R (4.0.5, with adegent (2.1.5), ape (5.6.2), and phenocamapi (0.1.5)), and MAFFT (7.520); 3. a high-performance computing environment on UC Berkeley's Savio compute cluster, including Scientific Linux (7.9), Bash (4.2.26), GDAL (2.2.3), Python (3.7, with numpy (1.21.5), rasterio (1.1.5), xarray (0.20.2), rioxarray (0.9.1), pandas (1.3.5), geopandas (0.8.1), json (2.0.9), tensorflow (2.3.1), affine (2.3.0), haversine (no version), eofs (1.4.0), scipy (1.4.1), sklearn (0.21.3), and matplotlib (3.1.1)), R (4.0.3, with dplyr (1.0.8), rgdal (1.5.18), sp (1.4.6), sf (0.9.7), raster (3.4.5), maps (3.4.0), rsample (0.1.1), ranger (0.13.1), Boruta (7.0.0), fastshap (0.0.7), vip (0.3.2), pdp (0.7.0), ggplot2 (3.3.5), ggthemes (4.2.4), grid (4.0.3), and RColorBrewer (1.1.2)), and Julia (1.4.1, with Distributed (no version), OrderedCollections (1.4.1), StaticArrays (1.3.5), Glob (1.3.0), TFRecord (0.1.0), JSON (0.21.3), ArchGDAL (0.7.4), Distances (0.10.7), NearestNeighbors (0.4.9), Statistics (no version), StatsBase (0.33.16), GLM (1.6.1), and Colors (0.12.8)).

All custom code and details of the computing environments used to run it are published in this project's GitHub repository (https://github.com/erthward/phen_asynch).

For manuscripts utilizing custom algorithms or software that are central to the research but not yet described in published literature, software must be made available to editors and reviewers. We strongly encourage code deposition in a community repository (e.g. GitHub). See the Nature Portfolio guidelines for submitting code & software for further information.

Data

Policy information about availability of data

All manuscripts must include a data availability statement. This statement should provide the following information, where applicable:

- Accession codes, unique identifiers, or web links for publicly available datasets
- A description of any restrictions on data availability
- For clinical datasets or third party data, please ensure that the statement adheres to our policy

Input datasets (Supplementary Table 1) are publicly available and were accessed using the following resources: MODIS MCD43A4 v061 surface reflectance (https://developers.google.com/earth-engine/datasets/catalog/MODIS_061_MCD43A4), OCO-2 sun-induced chlorophyll fluorescence (https://daac.ornl.gov/VEGETATION/guides/Global_High_Res_SIF_OCO2.html), TROPOMI SIF (https://doi.org/10.22002/D1.1347), MODIS MCD12C1.061 annual land cover (https://developers.google.com/earth-engine/datasets/catalog/MODIS_061_MCD12C1), PhenoCam normalized difference vegetation index (accessed using R package phenocamapi: https://github.com/PhenoCamNetwork/phenocamapi), FLUXNET gross primary productivity (https://fluxnet.org/data/), percent annual herbaceous cover in the Great Basin (https://doi.org/10.5066/P9VL3LD5), TerraClimate (https://developers.google.com/earth-engine/datasets/catalog/IDAHO_EPSCOR_TERRACLIMATE), MODIS Aqua and Terra surface reflectance cloud bands (https://developers.google.com/earth-engine/datasets/catalog/MODIS_061_MYD09GA, https://developers.google.com/earth-engine/datasets/catalog/MODIS_061_MOD09GA), EarthEnv topographic complexity (http://www.earthenv.org/topography), Global Land Analysis & Discovery global land cover and land use 2019 (https://glad.umd.edu/dataset/global-land-cover-land-use-v1), MODIS MCD64A1.v061 monthly burned area (https://developers.google.com/earth-engine/datasets/catalog/MODIS_061_MCD64A1), WorldClim bioclimatic variables (https://www.worldclim.org/data/worldclim21.html), iNaturalist flowering observations (accessed using Python package ipynaturalist, https://github.com/pyinat/pyinaturalist), Rhinella granulosa single nucleotide polymorphism data (https://datadryad.org/stash/dataset/doi:10.5061/dryad.pc866t1p4), Xiphorhynchus fuscus cytochrome B sequence data (http://zenodo.org/records/5012226), and Fedecafé Colombian coffee harvest season map data (digitized from https://doi.org/10.19053/20275137.3200). All data supporting the findings of this study are archived with Zenodo (DOI: https://doi.org/10.5281/zenodo.15654956) 127. A Google Earth Engine App provides the ability to explore the LSP modeling method, the global LSP map displayed in Fig. 1, and the global LSP asynchrony map displayed in Fig. 2A; it is demonstrated in Extended Data Fig. 2E and is linked in our GitHub repository (https://github.com/erthward/phen_asynch).

Research involving human participants, their data, or biological material

Policy information about studies with human participants or human data. See also policy information about sex, gender (identity/presentation), and sexual orientation and race, ethnicity and racism.

Reporting on sex and gender	not applicable
Reporting on race, ethnicity, or other socially relevant groupings	not applicable
Population characteristics	not applicable
Recruitment	not applicable
Ethics oversight	not applicable

Note that full information on the approval of the study protocol must also be provided in the manuscript.

Field-specific reporting

Please select the one below that is the best fit for your research. If you are not sure, read the appropriate sections before making your selection.

☐ Life sciences ☐ Behavioural & social sciences ☒ Ecological, evolutionary & environmental sciences

For a reference copy of the document with all sections, see nature.com/documents/nr-reporting-summary-flat.pdf

Life sciences study design

All studies must disclose on these points even when the disclosure is negative.

Sample size	*Describe how sample size was determined, detailing any statistical methods used to predetermine sample size OR if no sample-size calculation was performed, describe how sample sizes were chosen and provide a rationale for why these sample sizes are sufficient.*
Data exclusions	*Describe any data exclusions. If no data were excluded from the analyses, state so OR if data were excluded, describe the exclusions and the rationale behind them, indicating whether exclusion criteria were pre-established.*
Replication	*Describe the measures taken to verify the reproducibility of the experimental findings. If all attempts at replication were successful, confirm this OR if there are any findings that were not replicated or cannot be reproduced, note this and describe why.*
Randomization	*Describe how samples/organisms/participants were allocated into experimental groups. If allocation was not random, describe how covariates were controlled OR if this is not relevant to your study, explain why.*
Blinding	*Describe whether the investigators were blinded to group allocation during data collection and/or analysis. If blinding was not possible, describe why OR explain why blinding was not relevant to your study.*

Behavioural & social sciences study design

All studies must disclose on these points even when the disclosure is negative.

Study description	*Briefly describe the study type including whether data are quantitative, qualitative, or mixed-methods (e.g. qualitative cross-sectional, quantitative experimental, mixed-methods case study).*
Research sample	*State the research sample (e.g. Harvard university undergraduates, villagers in rural India) and provide relevant demographic information (e.g. age, sex) and indicate whether the sample is representative. Provide a rationale for the study sample chosen. For studies involving existing datasets, please describe the dataset and source.*
Sampling strategy	*Describe the sampling procedure (e.g. random, snowball, stratified, convenience). Describe the statistical methods that were used to predetermine sample size OR if no sample-size calculation was performed, describe how sample sizes were chosen and provide a rationale for why these sample sizes are sufficient. For qualitative data, please indicate whether data saturation was considered, and what criteria were used to decide that no further sampling was needed.*
Data collection	*Provide details about the data collection procedure, including the instruments or devices used to record the data (e.g. pen and paper, computer, eye tracker, video or audio equipment) whether anyone was present besides the participant(s) and the researcher, and whether the researcher was blind to experimental condition and/or the study hypothesis during data collection.*
Timing	*Indicate the start and stop dates of data collection. If there is a gap between collection periods, state the dates for each sample cohort.*
Data exclusions	*If no data were excluded from the analyses, state so OR if data were excluded, provide the exact number of exclusions and the rationale behind them, indicating whether exclusion criteria were pre-established.*
Non-participation	*State how many participants dropped out/declined participation and the reason(s) given OR provide response rate OR state that no participants dropped out/declined participation.*
Randomization	*If participants were not allocated into experimental groups, state so OR describe how participants were allocated to groups, and if allocation was not random, describe how covariates were controlled.*

Ecological, evolutionary & environmental sciences study design

All studies must disclose on these points even when the disclosure is negative.

Study description	We use remote sensing archives of MODIS near infrared reflectance of vegetation and sun-induced chlorophyll fluorescence to assess global patterns of land surface phenological diversity and spatial phenological asynchrony, to explore potential climatic and physiographic drivers of that asynchrony, and to test whether that asynchrony is more correlated with between-site climatic differences at higher latitudes than in the tropics. We then used iNaturalist flowering observation data, previously published genomic and genetic data, and a map of Colombian coffee harvest seasonality to demonstrate the utility of land surface phenology for

	predicting species-level spatial 'allochrony by allopatry', i.e., phenological asynchrony and genetic divergence as a function of the difference in seasonal timing between populations.
Research sample	The global land surface phenology datasets we present are derived from the MODIS land surface reflectance archive and (for validation of the MODIS data) a neural-net interpolated map of sun-induced chlorophyll fluorescence from the Orbiting Carbon Observatory 2. The samples in our global random forest analyses predicting the spatial asynchrony of land surface phenology are random samples chosen to represent our full global phenological dataset and quartile-stratified by the response variable. iNaturalist samples are opportunistic, voluntarily reported and annotated records of flowering, and genomic and genetic samples were determined by the designs of the studies from which they are derived.
Sampling strategy	In our random forest analyses, sample sizes (expressed as a fraction of the size of the full global dataset) was tuned as a hyperparameter during our model-development process (details described in methods). In our ensemble of permutation-based matrix regressions, sample sizes were up to 1000 points drawn within each high-asynchrony region (reduced due to dropout of any randomly drawn points that did not fall within valid pixels in the land surface phenology dataset). The samples in our ensemble of permutation-based matrix regressions are random samples of up to 1000 points drawn within the high-asynchrony regions defined by the regionalization algorithm described in the methods. Samples in the iNaturalist flowering phenological analysis included all available samples (as of the noted data of download) for all taxa passing a series of eligibility requirements. Samples for the genomic and genetic analyses derive from the only previously published genomic study of the Asynchrony of Seasons Hypothesis (ASH) and the only sympatric species among the few previously published genetic studies of the ASH. Samples for the Colombian coffee analysis were drawn across the entire area of a previously published map of coffee harvest seasonality.
Data collection	All input data comes from publicly available data archives and is documented in previous peer-reviewed publications.
Timing and spatial scale	Our main phenological dataset (MODIS near infrared reflectance of vegetation) provides data for every fourth day during the period from 2001/01/01 to 2020/12/31, providing us enough data to estimate the characteristic annual land surface phenology pattern for a location while remaining computable on Google Earth Engine. Our validation dataset (Orbiting Carbon Observatory 2-derived sun-induced chlorophyll fluorescence) provides twice-monthly data covering the period from 2014/09/01 to 2018/12/31, which was the longest available such archive at an acceptably high resolution at the time this study began.
Data exclusions	Pixels were excluded from the land surface phenology datasets if they contained >10% coverage of invalid land cover (e.g., permanent snow and ice, barren, or water bodies, where no true phenological pattern would be expected); if they were subject to land cover change that could contaminate the characteristic phenology being fitted (i.e., transitioned to/from agriculture); if they were missing >50% of potential data overall, >90% of data in any month (across years), or had a Pielou's data availability evenness of <0.8 (calculation described in methods), to prevent fitting of spurious phenological peaks during long, seasonally recurring periods of low data coverage, such as we observed in very high latitudes; or if permutation testing indicated that the R^2 of their fitted phenological harmonic regression was not significant (details provided in methods). To avoid sensitivity of downstream analyses to anthropogenic phenology patterns, all agricultural pixels were excluded from asynchrony maps and asynchrony-based analyses.
Reproducibility	Our results are not based on experiments, so experimental reproducibility could not be verified. However, we went to lengths to evaluate our findings against a second, independent remote sensing dataset, to assess the accuracy of that evaluation dataset itself, to tune the hyperparameters of our random forest models, and to cross-check our random forest modeling results across combinations of key' modeling decisions (choice between two response-variable datasets; choice of neighborhood within which to calculate pixels spatial asynchrony values; and choice of whether or not to include geographic coordinates as model features).
Randomization	Our study did not allocate units to groups. However, we used random subsampling when constructing predictive models on large, dense spatial datasets (details described in methods).
Blinding	Blinding was not relevant to our study, as sampling units were not assigned to treatments and controls.

Did the study involve field work? ☐ Yes ☒ No

Field work, collection and transport

Field conditions	*Describe the study conditions for field work, providing relevant parameters (e.g. temperature, rainfall).*
Location	*State the location of the sampling or experiment, providing relevant parameters (e.g. latitude and longitude, elevation, water depth).*
Access & import/export	*Describe the efforts you have made to access habitats and to collect and import/export your samples in a responsible manner and in compliance with local, national and international laws, noting any permits that were obtained (give the name of the issuing authority, the date of issue, and any identifying information).*
Disturbance	*Describe any disturbance caused by the study and how it was minimized.*

Reporting for specific materials, systems and methods

We require information from authors about some types of materials, experimental systems and methods used in many studies. Here, indicate whether each material, system or method listed is relevant to your study. If you are not sure if a list item applies to your research, read the appropriate section before selecting a response.

Materials & experimental systems

n/a	Involved in the study
☒	☐ Antibodies
☒	☐ Eukaryotic cell lines
☒	☐ Palaeontology and archaeology
☒	☐ Animals and other organisms
☒	☐ Clinical data
☒	☐ Dual use research of concern
☒	☐ Plants

Methods

n/a	Involved in the study
☒	☐ ChIP-seq
☒	☐ Flow cytometry
☒	☐ MRI-based neuroimaging

Antibodies

Antibodies used

Describe all antibodies used in the study; as applicable, provide supplier name, catalog number, clone name, and lot number.

Validation

Describe the validation of each primary antibody for the species and application, noting any validation statements on the manufacturer's website, relevant citations, antibody profiles in online databases, or data provided in the manuscript.

Eukaryotic cell lines

Policy information about cell lines and Sex and Gender in Research

Cell line source(s)

State the source of each cell line used and the sex of all primary cell lines and cells derived from human participants or vertebrate models.

Authentication

Describe the authentication procedures for each cell line used OR declare that none of the cell lines used were authenticated.

Mycoplasma contamination

Confirm that all cell lines tested negative for mycoplasma contamination OR describe the results of the testing for mycoplasma contamination OR declare that the cell lines were not tested for mycoplasma contamination.

Commonly misidentified lines
(See ICLAC register)

Name any commonly misidentified cell lines used in the study and provide a rationale for their use.

Palaeontology and Archaeology

Specimen provenance

Provide provenance information for specimens and describe permits that were obtained for the work (including the name of the issuing authority, the date of issue, and any identifying information). Permits should encompass collection and, where applicable, export.

Specimen deposition

Indicate where the specimens have been deposited to permit free access by other researchers.

Dating methods

If new dates are provided, describe how they were obtained (e.g. collection, storage, sample pretreatment and measurement), where they were obtained (i.e. lab name), the calibration program and the protocol for quality assurance OR state that no new dates are provided.

☐ Tick this box to confirm that the raw and calibrated dates are available in the paper or in Supplementary Information.

Ethics oversight

Identify the organization(s) that approved or provided guidance on the study protocol, OR state that no ethical approval or guidance was required and explain why not.

Note that full information on the approval of the study protocol must also be provided in the manuscript.

Animals and other research organisms

Policy information about studies involving animals; ARRIVE guidelines recommended for reporting animal research, and Sex and Gender in Research

Laboratory animals

For laboratory animals, report species, strain and age OR state that the study did not involve laboratory animals.

Wild animals

Provide details on animals observed in or captured in the field; report species and age where possible. Describe how animals were caught and transported and what happened to captive animals after the study (if killed, explain why and describe method; if released, say where and when) OR state that the study did not involve wild animals.

Reporting on sex

Indicate if findings apply to only one sex; describe whether sex was considered in study design, methods used for assigning sex. Provide data disaggregated for sex where this information has been collected in the source data as appropriate; provide overall

	numbers in this Reporting Summary. Please state if this information has not been collected. Report sex-based analyses where performed, justify reasons for lack of sex-based analysis.
Field-collected samples	*For laboratory work with field-collected samples, describe all relevant parameters such as housing, maintenance, temperature, photoperiod and end-of-experiment protocol OR state that the study did not involve samples collected from the field.*
Ethics oversight	*Identify the organization(s) that approved or provided guidance on the study protocol, OR state that no ethical approval or guidance was required and explain why not.*

Note that full information on the approval of the study protocol must also be provided in the manuscript.

Clinical data

Policy information about clinical studies

All manuscripts should comply with the ICMJE guidelines for publication of clinical research and a completed CONSORT checklist must be included with all submissions.

Clinical trial registration	*Provide the trial registration number from ClinicalTrials.gov or an equivalent agency.*
Study protocol	*Note where the full trial protocol can be accessed OR if not available, explain why.*
Data collection	*Describe the settings and locales of data collection, noting the time periods of recruitment and data collection.*
Outcomes	*Describe how you pre-defined primary and secondary outcome measures and how you assessed these measures.*

Dual use research of concern

Policy information about dual use research of concern

Hazards

Could the accidental, deliberate or reckless misuse of agents or technologies generated in the work, or the application of information presented in the manuscript, pose a threat to:

No	Yes	
☐	☐	Public health
☐	☐	National security
☐	☐	Crops and/or livestock
☐	☐	Ecosystems
☐	☐	Any other significant area

Experiments of concern

Does the work involve any of these experiments of concern:

No	Yes	
☐	☐	Demonstrate how to render a vaccine ineffective
☐	☐	Confer resistance to therapeutically useful antibiotics or antiviral agents
☐	☐	Enhance the virulence of a pathogen or render a nonpathogen virulent
☐	☐	Increase transmissibility of a pathogen
☐	☐	Alter the host range of a pathogen
☐	☐	Enable evasion of diagnostic/detection modalities
☐	☐	Enable the weaponization of a biological agent or toxin
☐	☐	Any other potentially harmful combination of experiments and agents

Plants

Seed stocks	not applicable
Novel plant genotypes	not applicable
Authentication	not applicable

ChIP-seq

Data deposition

☐ Confirm that both raw and final processed data have been deposited in a public database such as GEO.

☐ Confirm that you have deposited or provided access to graph files (e.g. BED files) for the called peaks.

Data access links<br>*May remain private before publication.*	*For "Initial submission" or "Revised version" documents, provide reviewer access links. For your "Final submission" document, provide a link to the deposited data.*
Files in database submission	*Provide a list of all files available in the database submission.*
Genome browser session<br>(e.g. UCSC)	*Provide a link to an anonymized genome browser session for "Initial submission" and "Revised version" documents only, to enable peer review. Write "no longer applicable" for "Final submission" documents.*

Methodology

Replicates	*Describe the experimental replicates, specifying number, type and replicate agreement.*
Sequencing depth	*Describe the sequencing depth for each experiment, providing the total number of reads, uniquely mapped reads, length of reads and whether they were paired- or single-end.*
Antibodies	*Describe the antibodies used for the ChIP-seq experiments; as applicable, provide supplier name, catalog number, clone name, and lot number.*
Peak calling parameters	*Specify the command line program and parameters used for read mapping and peak calling, including the ChIP, control and index files used.*
Data quality	*Describe the methods used to ensure data quality in full detail, including how many peaks are at FDR 5% and above 5-fold enrichment.*
Software	*Describe the software used to collect and analyze the ChIP-seq data. For custom code that has been deposited into a community repository, provide accession details.*

Flow Cytometry

Plots

Confirm that:

☐ The axis labels state the marker and fluorochrome used (e.g. CD4-FITC).

☐ The axis scales are clearly visible. Include numbers along axes only for bottom left plot of group (a 'group' is an analysis of identical markers).

☐ All plots are contour plots with outliers or pseudocolor plots.

☐ A numerical value for number of cells or percentage (with statistics) is provided.

Methodology

Sample preparation	*Describe the sample preparation, detailing the biological source of the cells and any tissue processing steps used.*
Instrument	*Identify the instrument used for data collection, specifying make and model number.*
Software	*Describe the software used to collect and analyze the flow cytometry data. For custom code that has been deposited into a community repository, provide accession details.*

Cell population abundance | *Describe the abundance of the relevant cell populations within post-sort fractions, providing details on the purity of the samples and how it was determined.*

Gating strategy | *Describe the gating strategy used for all relevant experiments, specifying the preliminary FSC/SSC gates of the starting cell population, indicating where boundaries between "positive" and "negative" staining cell populations are defined.*

☐ Tick this box to confirm that a figure exemplifying the gating strategy is provided in the Supplementary Information.

Magnetic resonance imaging

Experimental design

Design type | *Indicate task or resting state; event-related or block design.*

Design specifications | *Specify the number of blocks, trials or experimental units per session and/or subject, and specify the length of each trial or block (if trials are blocked) and interval between trials.*

Behavioral performance measures | *State number and/or type of variables recorded (e.g. correct button press, response time) and what statistics were used to establish that the subjects were performing the task as expected (e.g. mean, range, and/or standard deviation across subjects).*

Acquisition

Imaging type(s) | *Specify: functional, structural, diffusion, perfusion.*

Field strength | *Specify in Tesla*

Sequence & imaging parameters | *Specify the pulse sequence type (gradient echo, spin echo, etc.), imaging type (EPI, spiral, etc.), field of view, matrix size, slice thickness, orientation and TE/TR/flip angle.*

Area of acquisition | *State whether a whole brain scan was used OR define the area of acquisition, describing how the region was determined.*

Diffusion MRI ☐ Used ☐ Not used

Preprocessing

Preprocessing software | *Provide detail on software version and revision number and on specific parameters (model/functions, brain extraction, segmentation, smoothing kernel size, etc.).*

Normalization | *If data were normalized/standardized, describe the approach(es): specify linear or non-linear and define image types used for transformation OR indicate that data were not normalized and explain rationale for lack of normalization.*

Normalization template | *Describe the template used for normalization/transformation, specifying subject space or group standardized space (e.g. original Talairach, MNI305, ICBM152) OR indicate that the data were not normalized.*

Noise and artifact removal | *Describe your procedure(s) for artifact and structured noise removal, specifying motion parameters, tissue signals and physiological signals (heart rate, respiration).*

Volume censoring | *Define your software and/or method and criteria for volume censoring, and state the extent of such censoring.*

Statistical modeling & inference

Model type and settings | *Specify type (mass univariate, multivariate, RSA, predictive, etc.) and describe essential details of the model at the first and second levels (e.g. fixed, random or mixed effects; drift or auto-correlation).*

Effect(s) tested | *Define precise effect in terms of the task or stimulus conditions instead of psychological concepts and indicate whether ANOVA or factorial designs were used.*

Specify type of analysis: ☐ Whole brain ☐ ROI-based ☐ Both

Statistic type for inference (See Eklund et al. 2016) | *Specify voxel-wise or cluster-wise and report all relevant parameters for cluster-wise methods.*

Correction | *Describe the type of correction and how it is obtained for multiple comparisons (e.g. FWE, FDR, permutation or Monte Carlo).*

Models & analysis

n/a	Involved in the study
☐	☐ Functional and/or effective connectivity
☐	☐ Graph analysis
☐	☐ Multivariate modeling or predictive analysis

Functional and/or effective connectivity	*Report the measures of dependence used and the model details (e.g. Pearson correlation, partial correlation, mutual information).*
Graph analysis	*Report the dependent variable and connectivity measure, specifying weighted graph or binarized graph, subject- or group-level, and the global and/or node summaries used (e.g. clustering coefficient, efficiency, etc.).*
Multivariate modeling and predictive analysis	*Specify independent variables, features extraction and dimension reduction, model, training and evaluation metrics.*
